# Supplementary material for: Novel Anthraquinone-Based Benzenesulfonamide Derivatives and Their Analogues as Potent Human Carbonic Anhydrase Inhibitors with Antitumor Activity: Synthesis, Biological Evaluation, and In Silico Analysis
Source: Int J Mol Sci. 2024 Mar 15;25(6):3348. doi: 10.3390/ijms25063348 (PMC10969960; doi:10.3390/ijms25063348)
Supplement: Supplementary file 1 [file ijms-25-03348-s001.zip › ijms-2867233-supplementary.pdf]

Supplementary materials

Novel Anthraquinone–Based Benzenesulfonamide Derivatives and Their Analogues as Potent Human Carbonic Anhydrase Inhibitors with Antitumor Activity: Synthesis, Biological Evaluation, and In Silico Analysis

Contents

1. Figure S1

2. Tables S1and S2

3. ^{1}H-NMR, ^{13}C-NMR and HRMS Spectra data S1–S23

4. HPLC data S24

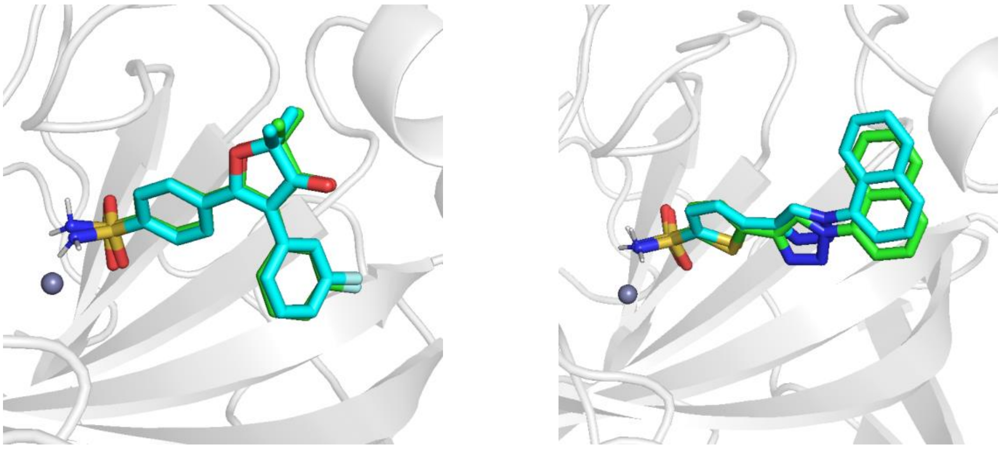

(**a**) (**b**)

Figure S1. Result of redocking. (a) Crystal conformation of CA II and original ligand (cyan) and docked conformation (green); (b) Crystal conformation of CA IX and original ligand (cyan) and docked conformation (green).

Table S1. The docking score, binding interactions, and IC_{50} of the active compounds in *h*CA II enzyme

Compound	Docking score (Kcal/mol)	Amino acid interactions	Interacting groups	IC_{50} (nM)
**5c**	-12.98	Zn^{2+} (metal)<br>T198 (H-bond)<br>F130 (pi-stacking)<br>E69 (hydrophobic)<br>I91 (hydrophobic)<br>Q92 (hydrophobic)<br>V121 (hydrophobic)<br>L197 (hydrophobic)	NH of SO_2NH_2<br>NH and O of SO_2NH_2<br>Anthraquinone ring	41.58
**5h**	-13.17	Zn^{2+} (metal)<br>N67 (H-bond)<br>Q92 (H-bond)<br>T198 (H-bond)<br>F130 (pi-stacking)<br>L60 (hydrophobic)<br>L91 (hydrophobic)<br>V121 (hydrophobic)<br>L140 (hydrophobic)<br>V142 (hydrophobic)<br>L197 (hydrophobic)	NH of SO_2NH_2<br>O of anthraquinone ring<br>O of anthraquinone ring<br>NH and O of SO_2NH_2<br>Anthraquinone ring	21.19
**6c**	-12.83	Zn^{2+} (metal)<br>T198 (H-bond)<br>T199 (H-bond)<br>F130 (pi-stacking)<br>E69 (hydrophobic)<br>I91 (hydrophobic)<br>Q92 (hydrophobic)<br>L197 (hydrophobic)	NH of SO_2NH_2<br>NH and O of SO_2NH_2<br>O of SO_2NH_2<br>Anthraquinone ring	68.34
**5k**	-9.97	N67 (H-bond)<br>Q92 (H-bond)<br>T199 (H-bond)<br>H94 (pi-stacking)<br>F130 (pi-stacking)<br>GLU69 (hydrophobic)<br>GLN92 (hydrophobic)<br>VAL121 (hydrophobic)<br>LEU140 (hydrophobic)<br>VAL142 (hydrophobic)<br>LEU197 (hydrophobic)<br>THR198 (hydrophobic)<br>TRP208 (hydrophobic)	O of SO_2NH<br>O of SO_2NH<br>O of anthraquinone ring<br>Anthraquinone ring<br>Benzene ring	155.91

Table S2. The docking score, binding interactions, and IC_{50} of the active compounds in *h*CA IX enzyme

Compound	Docking score (Kcal/mol)	Amino acid interactions	Interacting groups	IC_{50} (nM)
**5c**	-13.08	Zn^{2+} (metal)<br>W9 (H-bond)<br>E106 (H-bond)<br>T200 (H-bond)<br>T201 (H-bond)<br>P202 (H-bond)<br>V20 (hydrophobic)<br>L199 (hydrophobic)<br>P202 (hydrophobic)<br>P203 (hydrophobic)	NH of SO_2NH_2<br>O of amide<br>NH of SO_2NH_2<br>O of SO_2NH_2<br>NH of amide<br>NH of amide	40.58
**5h**	-13.19	Zn^{2+} (metal)<br>W9 (H-bond)<br>E106 (H-bond)<br>T200 (H-bond)<br>P202 (H-bond)<br>V20 (hydrophobic)<br>L199 (hydrophobic)<br>P202 (hydrophobic)<br>P203 (hydrophobic)	NH of SO_2NH_2<br>O of amide<br>NH of SO_2NH_2<br>O of SO_2NH_2<br>NH of amide	46.19
**6c**	-12.06	Zn^{2+} (metal)<br>Q92 (H-bond)<br>E106 (H-bond)<br>T200 (H-bond)<br>V121 (hydrophobic)<br>V130 (hydrophobic)<br>D131 (hydrophobic)<br>L134 (hydrophobic)<br>L199 (hydrophobic)	NH of SO_2NH_2<br>NH of amide<br>NH of SO_2NH_2<br>O of SO_2NH_2	34.88
**5k**	-9.46	W9 (H-bond)<br>T200 (H-bond)<br>T201 (H-bond)<br>P202 (H-bond)<br>H94 (pi-stacking)<br>W9 (hydrophobic)<br>H119 (hydrophobic)<br>V121 (hydrophobic)<br>V142 (hydrophobic)<br>L199 (hydrophobic)<br>P203 (hydrophobic)<br>W210 (hydrophobic)	O of amide<br>O of anthraquinone ring<br>O of anthraquinone ring<br>NH of amide<br>Anthraquinone ring	226.58

Data S1. ^{1}H-NMR, ^{13}C-NMR and HRMS data of compound 5a

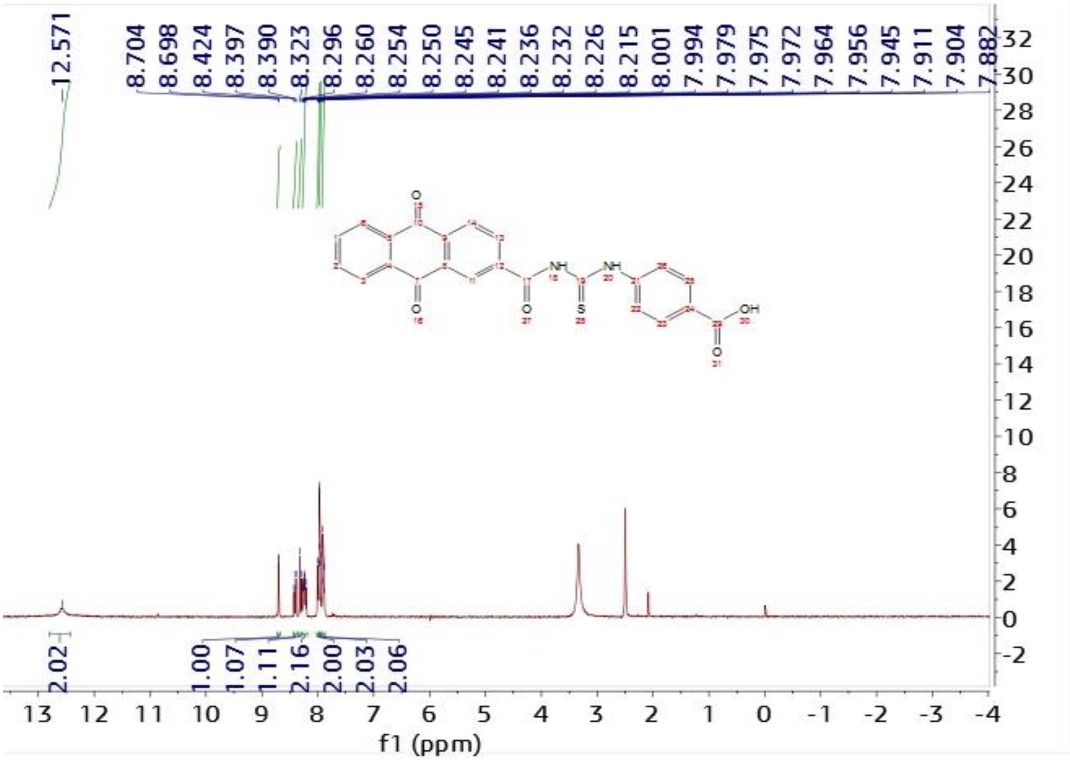

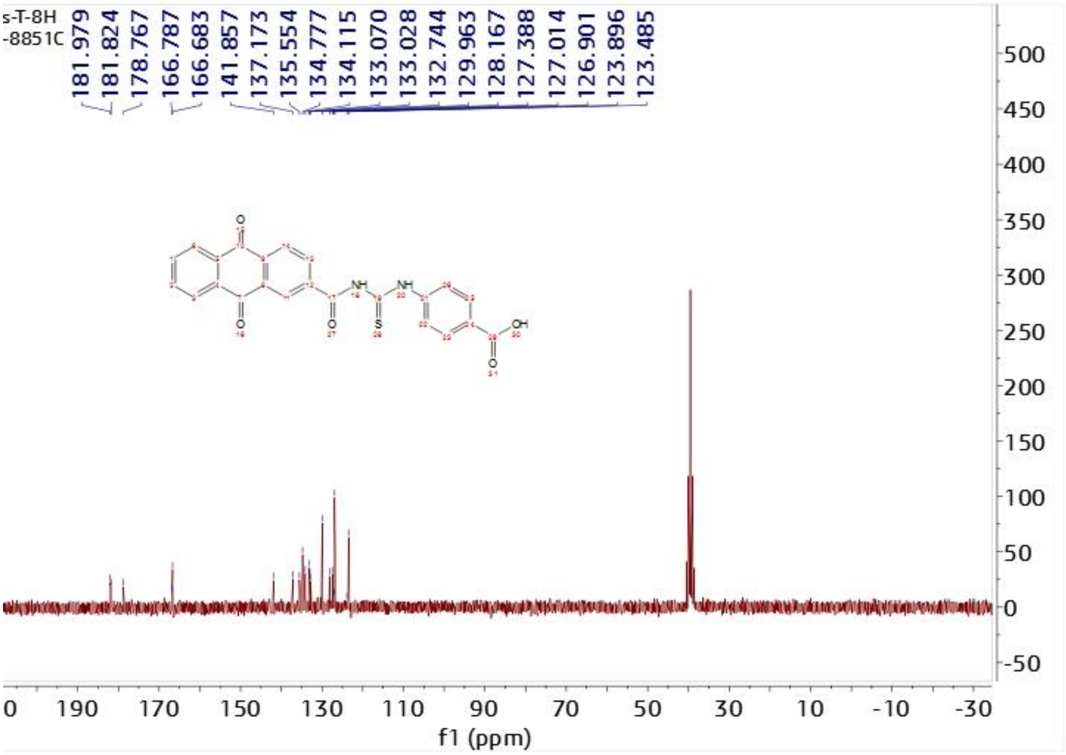

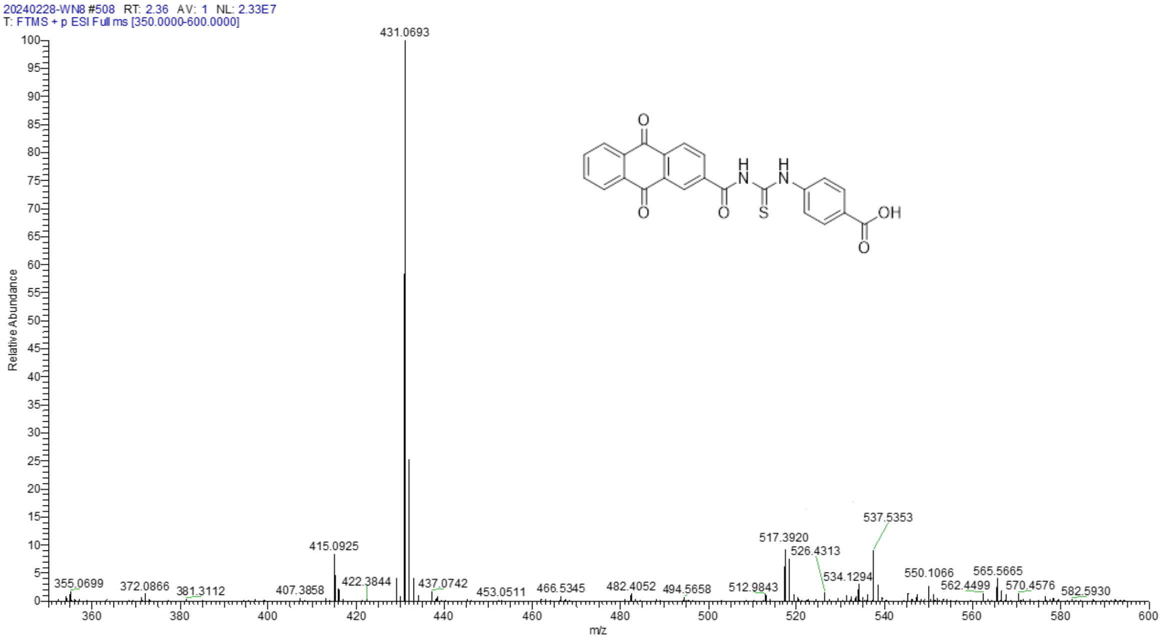

Data S2. ^{1}H-NMR, ^{13}C-NMR and HRMS data of compound 5b

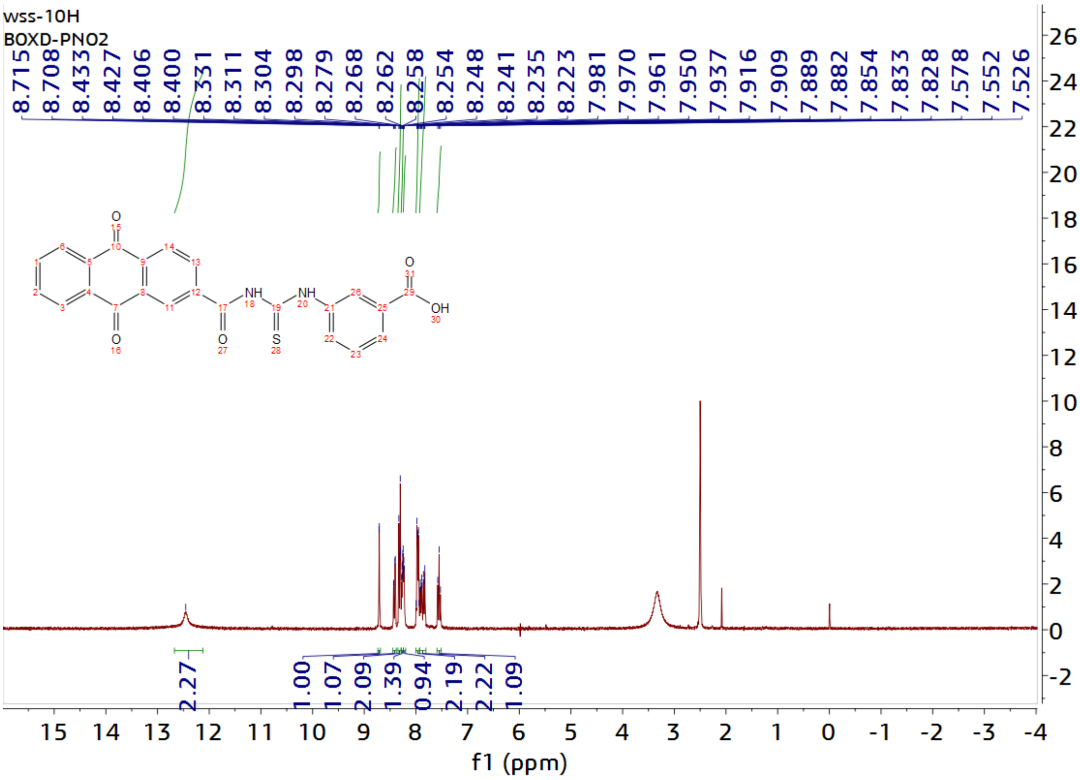

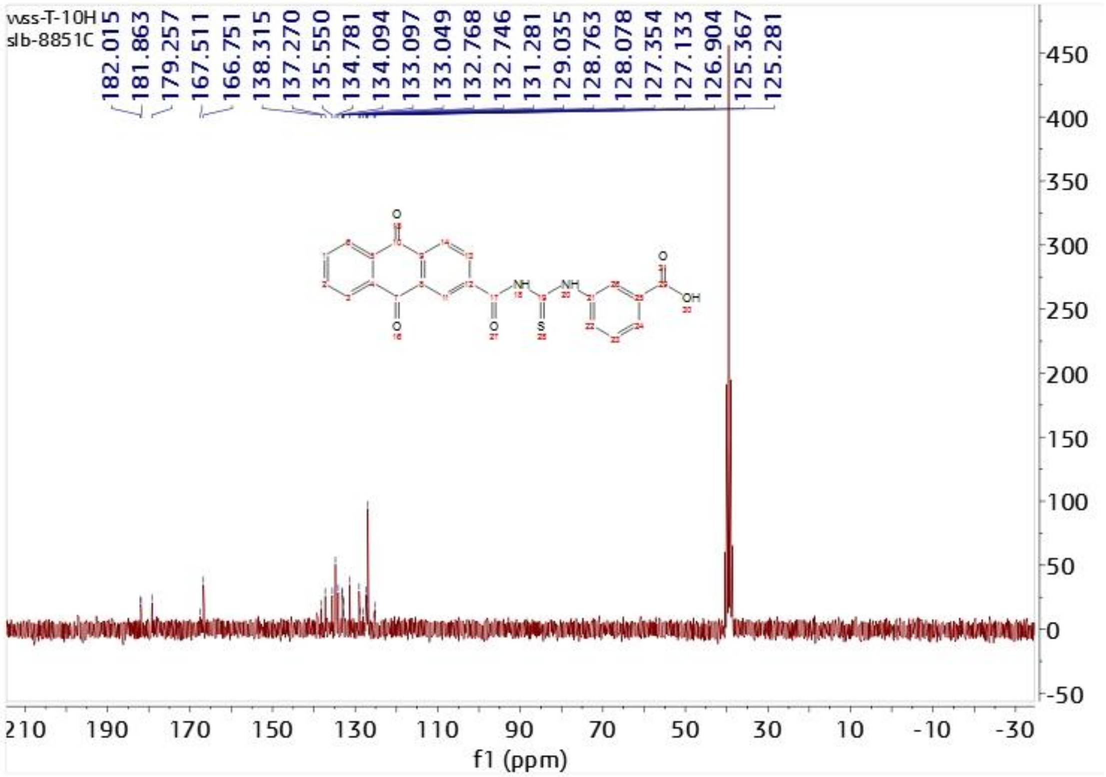

wss-T-10H
slb-8851C
182.015
181.863
179.257
167.511
166.751
138.315
137.270
135.550
134.781
134.094
133.097
133.049
132.768
132.746
131.281
129.035
128.763
128.078
127.354
127.133
126.904
125.367
125.281
f1 (ppm)

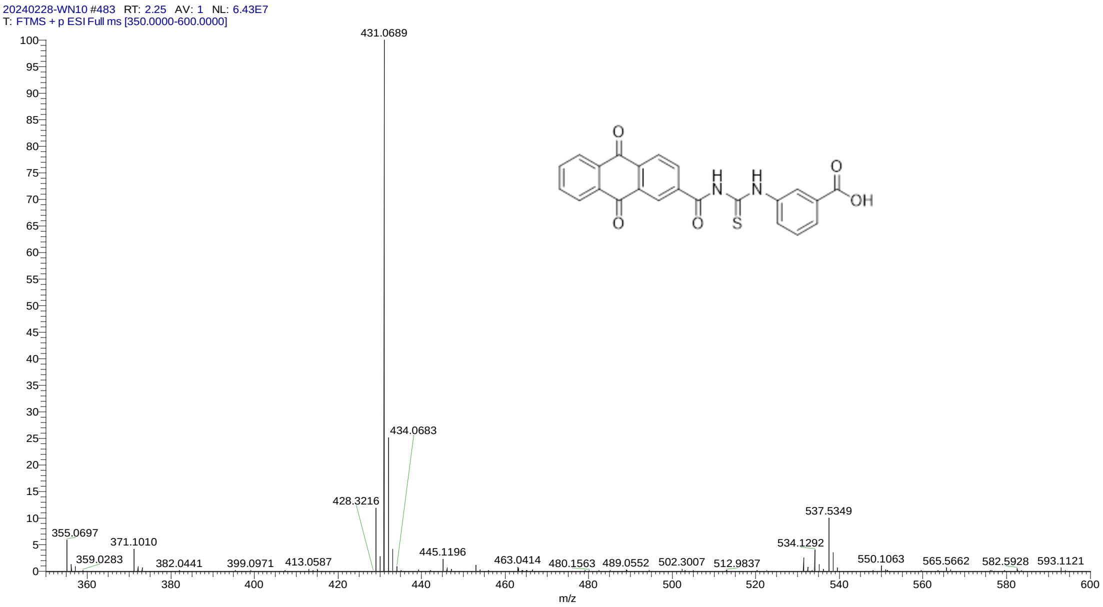

20240228-WN10 #483 RT: 2.25 AV: 1 NL: 6.43E7
T: FTMS + p ESI Full ms [350.0000-600.0000]
431.0689
434.0683
428.3216
537.5349
534.1292
355.0697
359.0283
371.1010
382.0441
399.0971
413.0587
445.1196
463.0414
480.1563
489.0552
502.3007
512.9837
550.1063
565.5662
582.5928
593.1121
m/z

date S3. ¹H-NMR, ¹³C-NMR and HRMS data of compound 5c

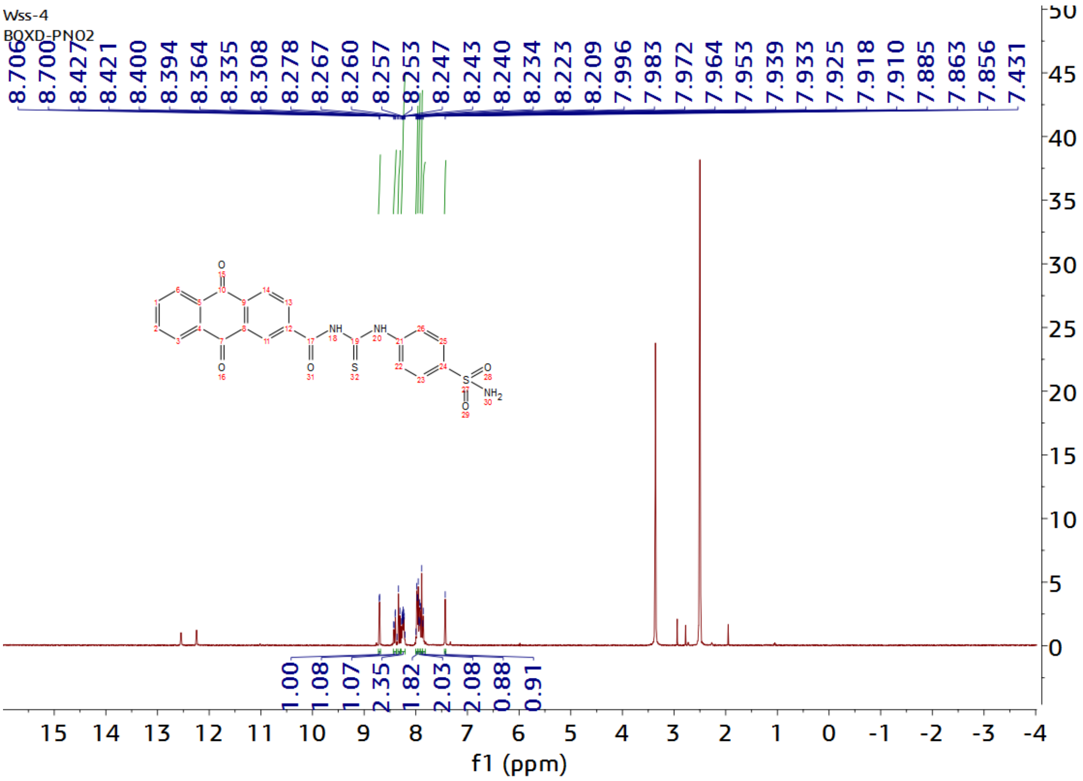

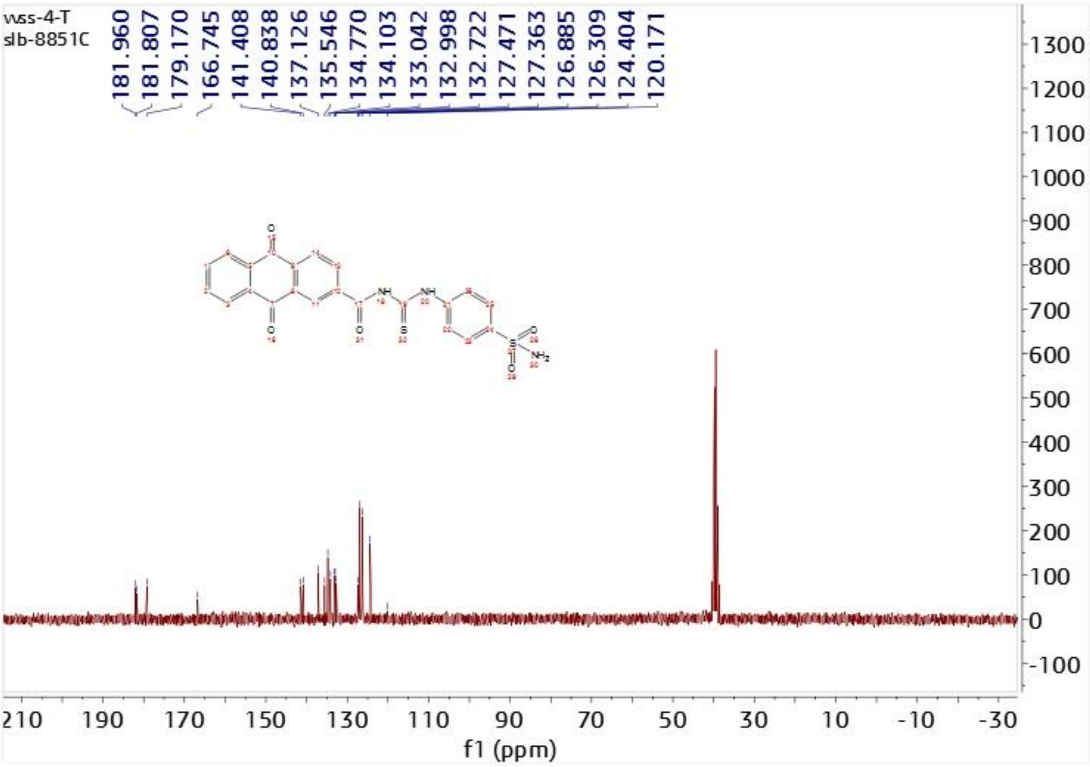

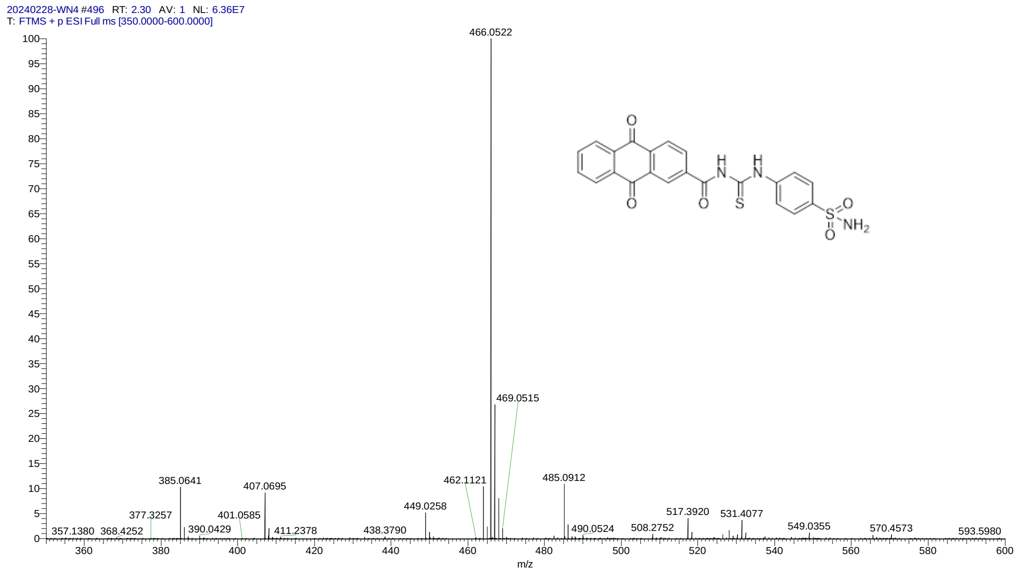

Data S4. ^{1}H-NMR, ^{13}C-NMR and HRMS data of compound 5d

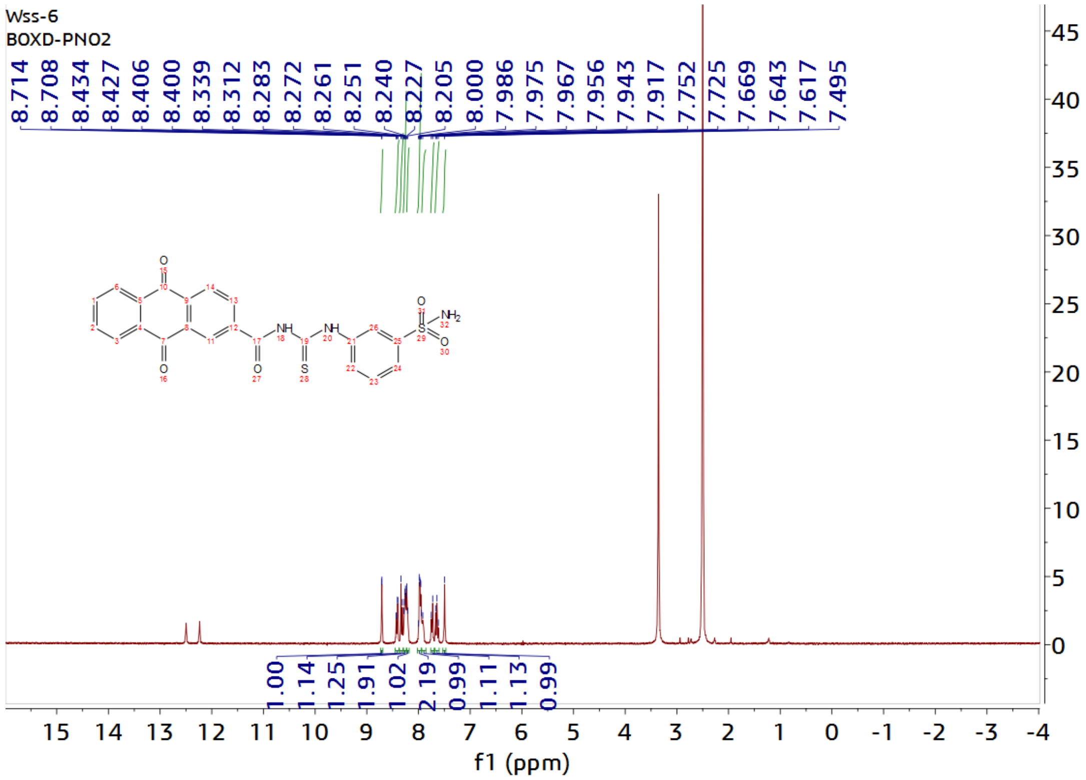

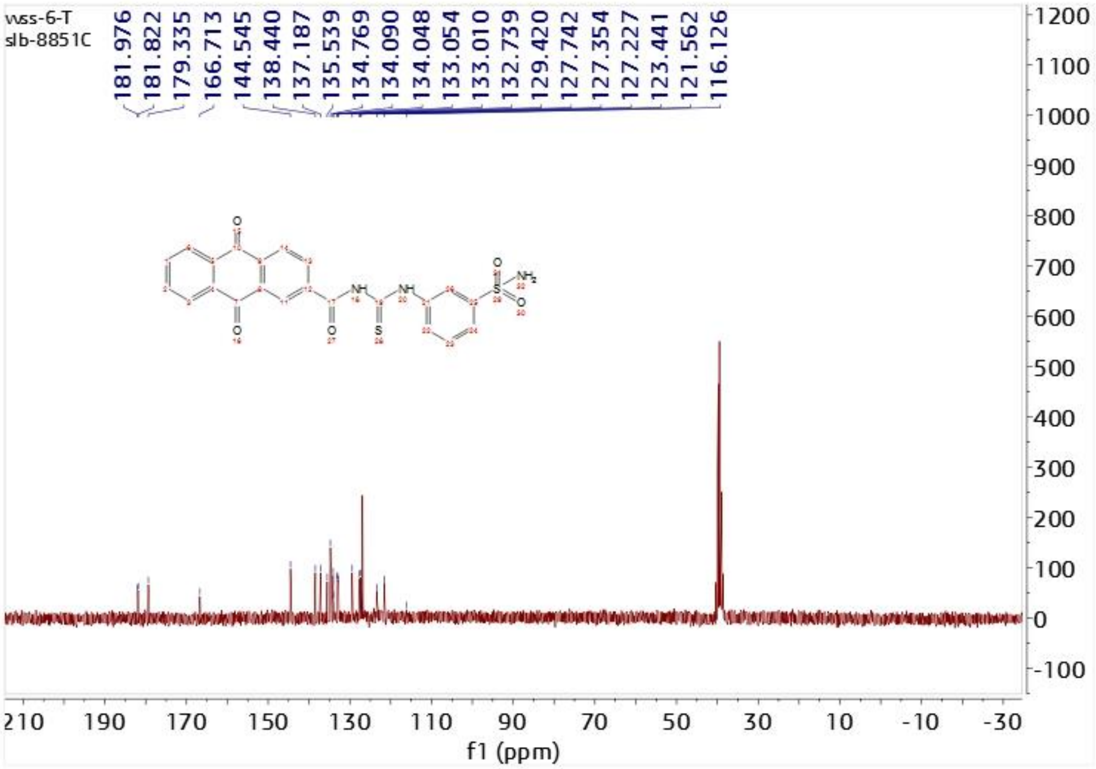

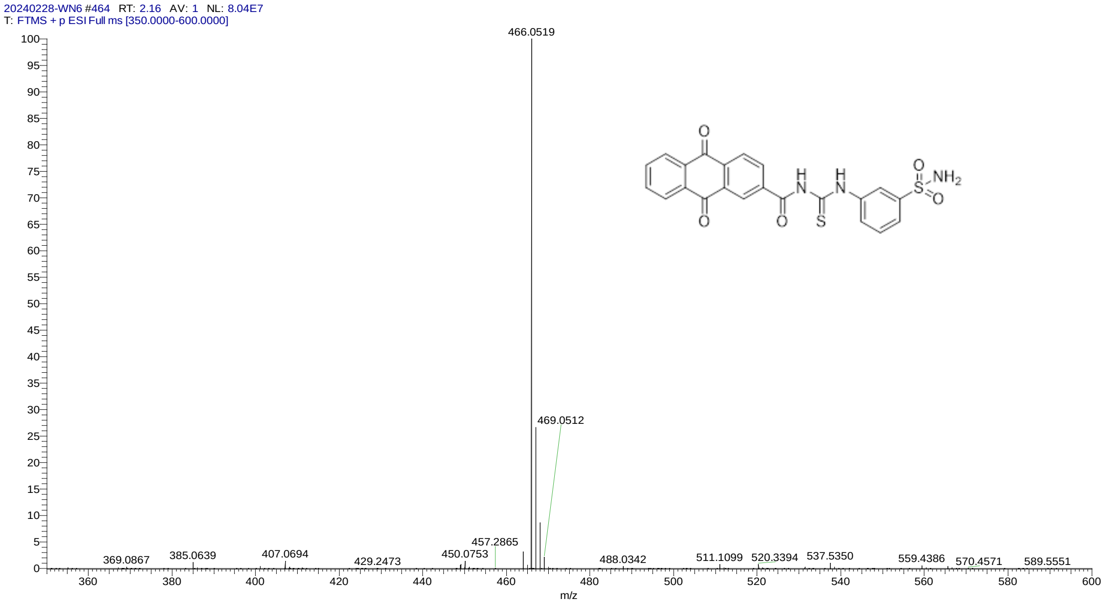

Data S5. ^{1}H-NMR, ^{13}C-NMR and HRMS data of compound 5e

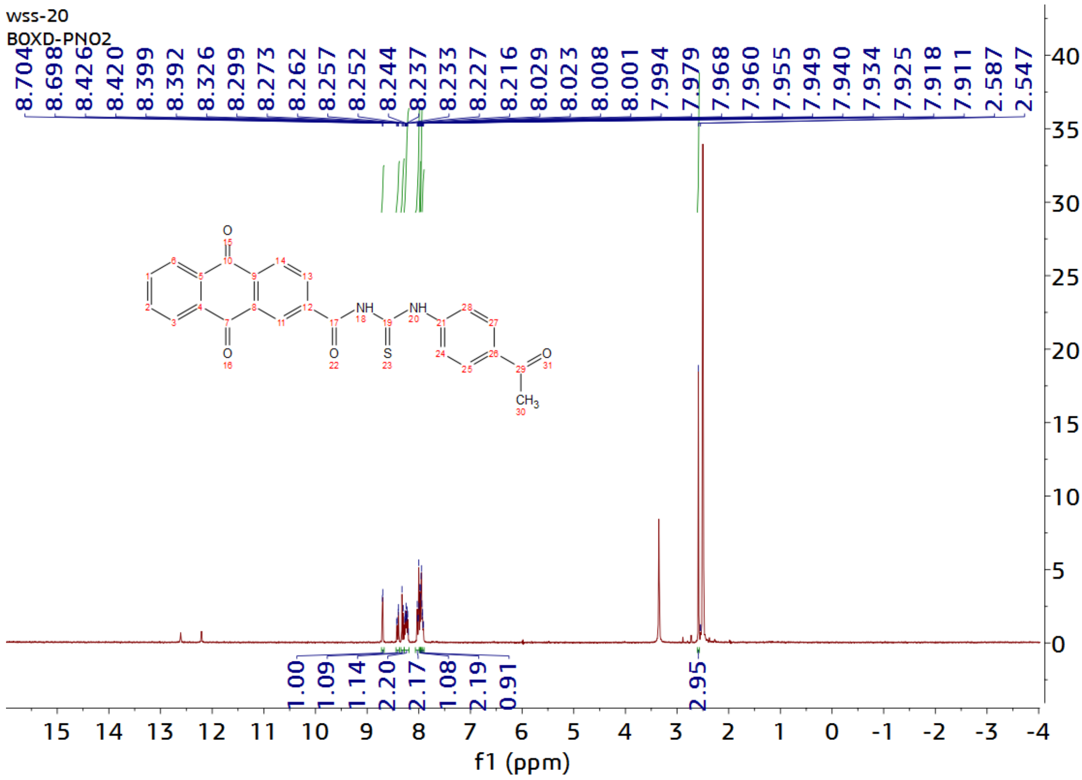
wss-20
BOXD-PNO2
8.704
8.698
8.426
8.420
8.399
8.392
8.326
8.299
8.273
8.262
8.257
8.252
8.244
8.237
8.233
8.227
8.216
8.029
8.023
8.008
8.001
7.994
7.979
7.968
7.960
7.955
7.949
7.940
7.934
7.925
7.918
7.911
2.587
2.547
1.00
1.09
1.14
2.20
2.17
1.08
2.19
0.91
2.95
f1 (ppm)

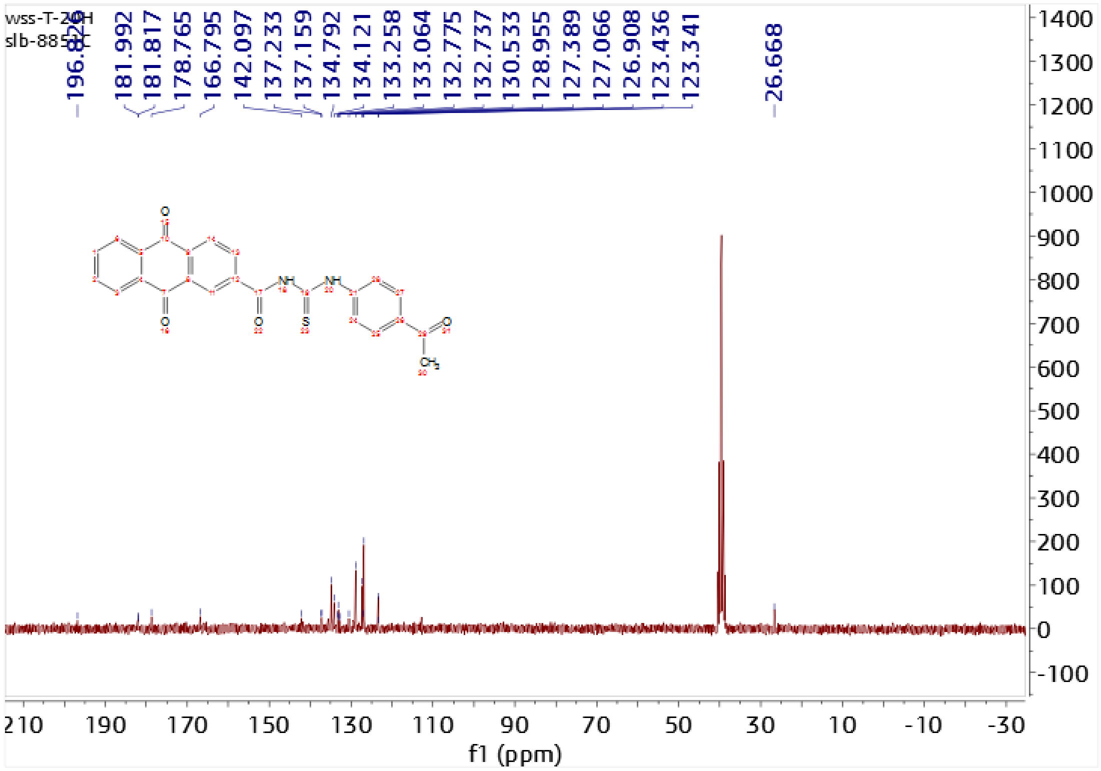
wss-T-20H
slb-885-C
196.826
181.992
181.817
178.765
166.795
142.097
137.233
137.159
134.792
134.121
133.258
133.064
132.775
132.737
130.533
128.955
127.389
127.066
126.908
123.436
123.341
26.668
f1 (ppm)

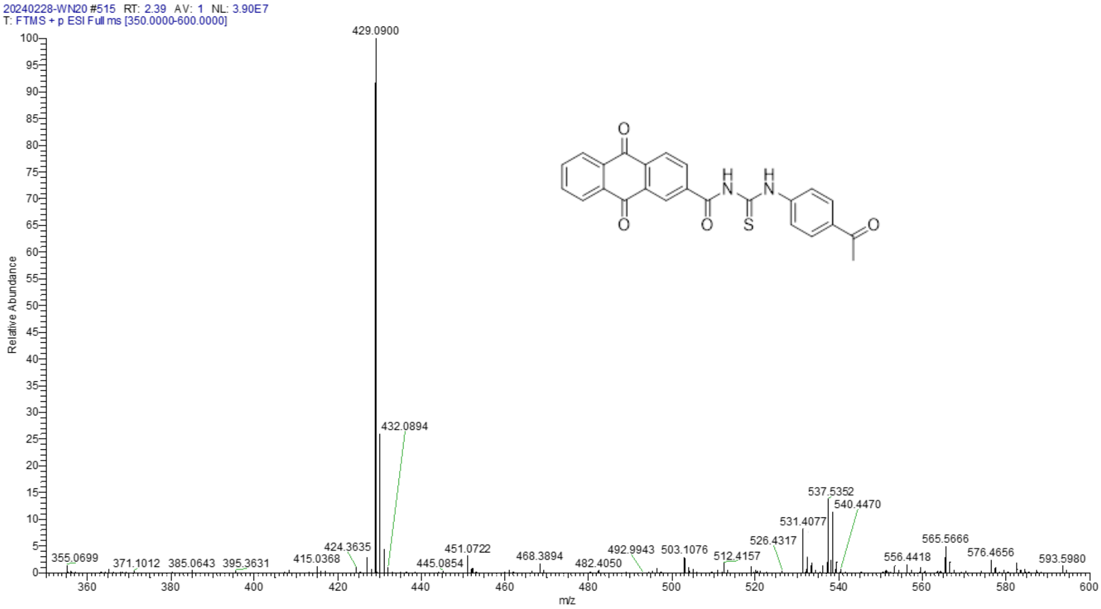

Data S6. ^{1}H-NMR, ^{13}C-NMR and HRMS data of compound 5f

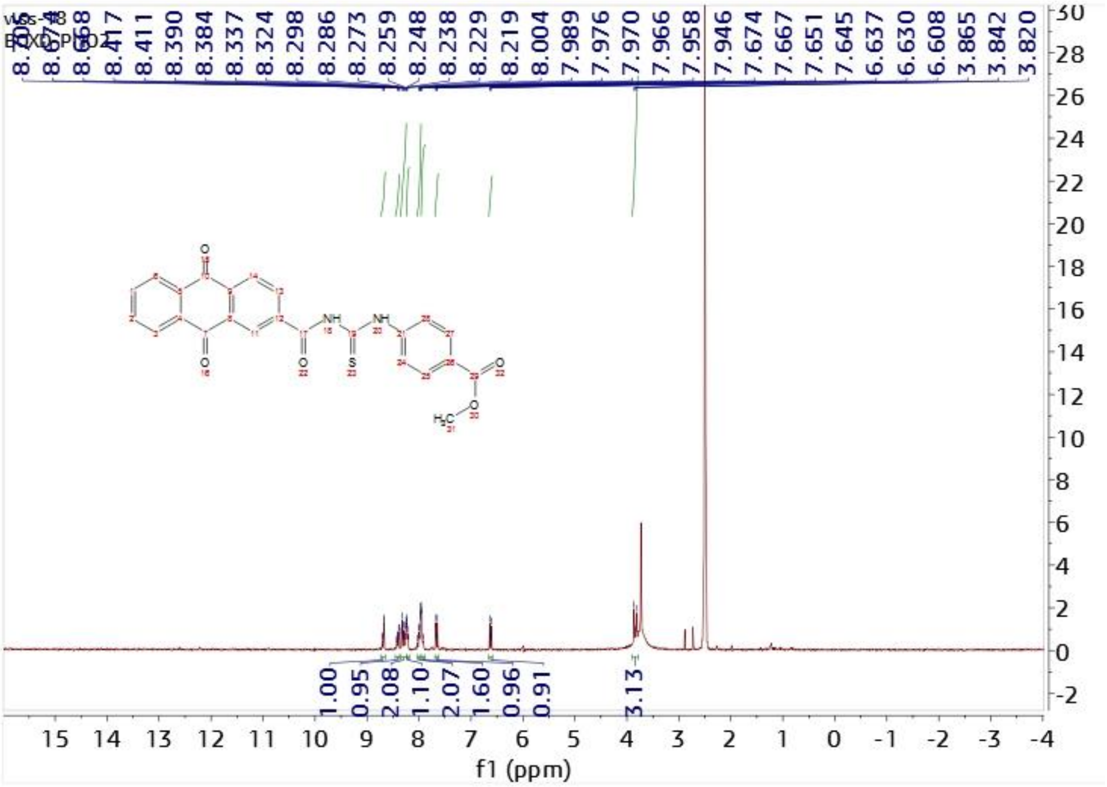

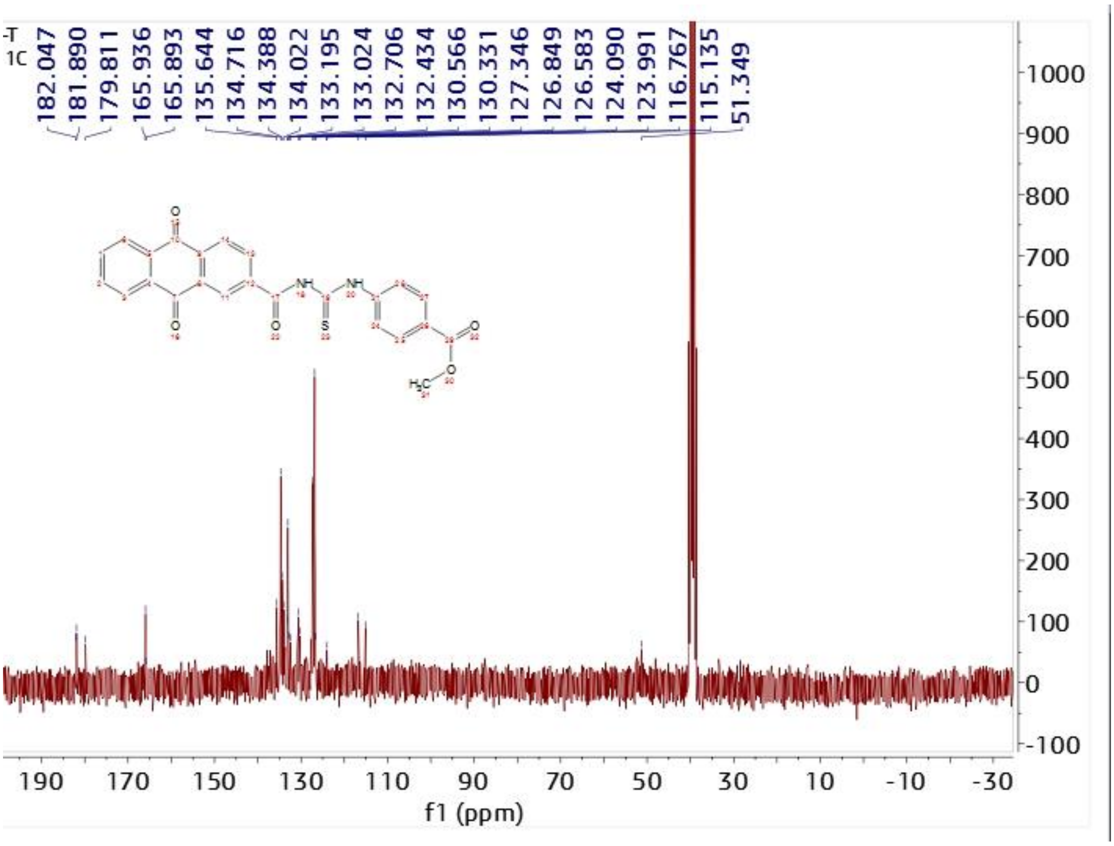

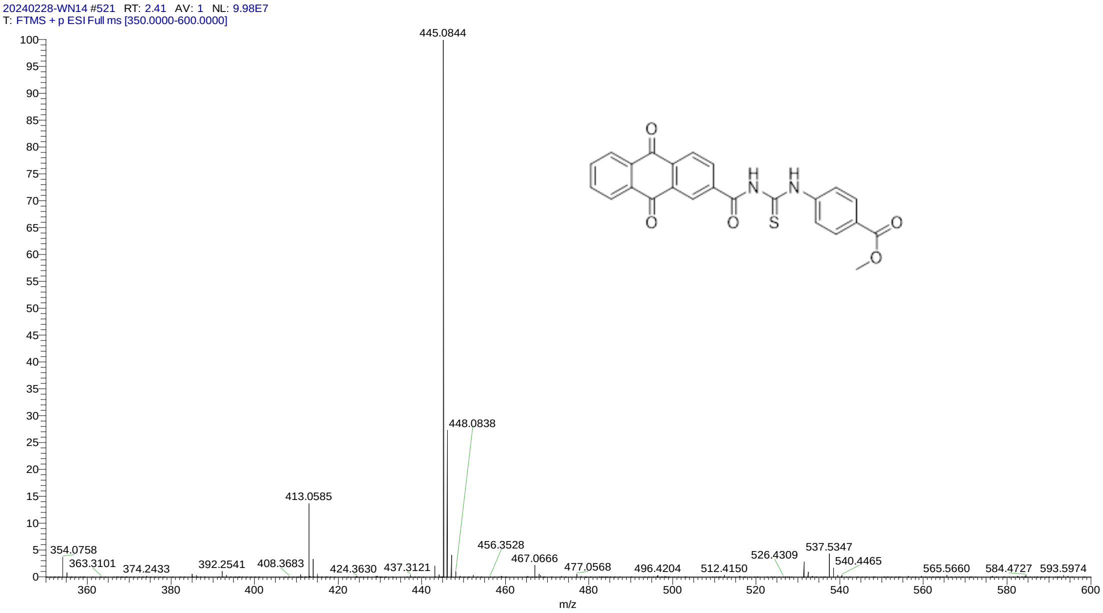

Data S7. ^{1}H-NMR, ^{13}C-NMR and HRMS data of compound 5g

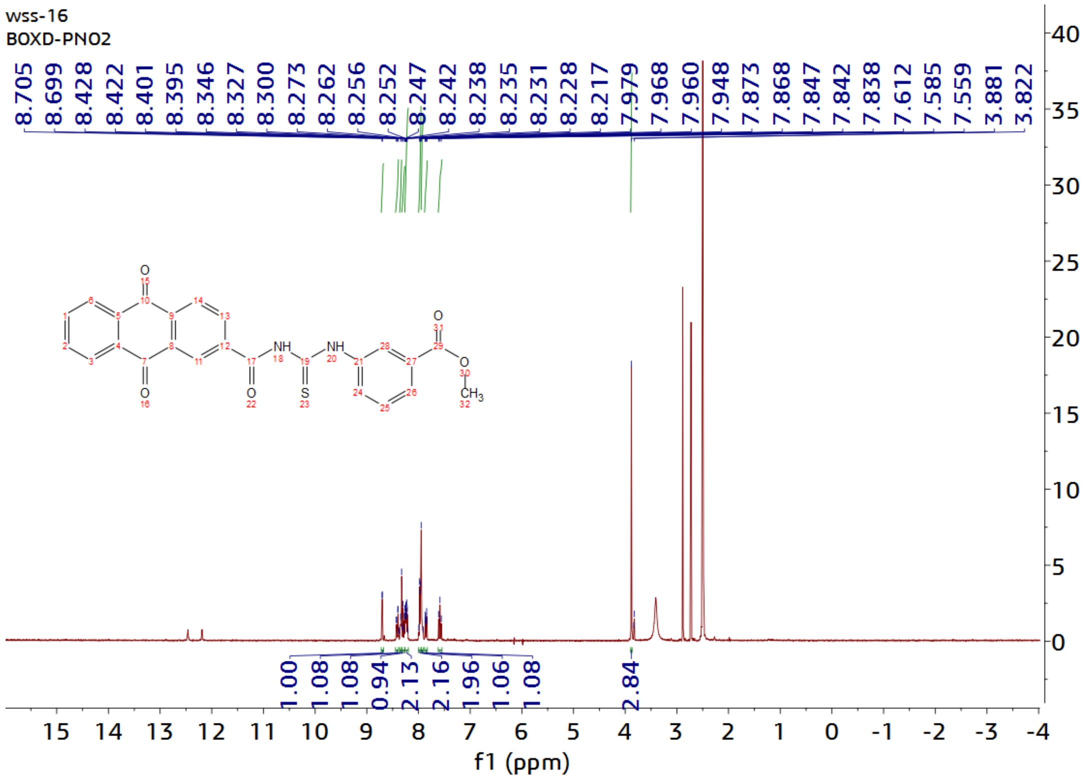
wss-16
BOXD-PNO2
8.705 8.699 8.428 8.422 8.401 8.395 8.346 8.327 8.300 8.273 8.262 8.256 8.252 8.247 8.242 8.238 8.235 8.231 8.228 8.217 7.979 7.968 7.960 7.948 7.873 7.868 7.847 7.842 7.838 7.612 7.585 7.559 3.881 3.822
1.00 1.08 1.08 0.94 2.13 2.16 1.96 1.06 1.08 2.84
f1 (ppm)

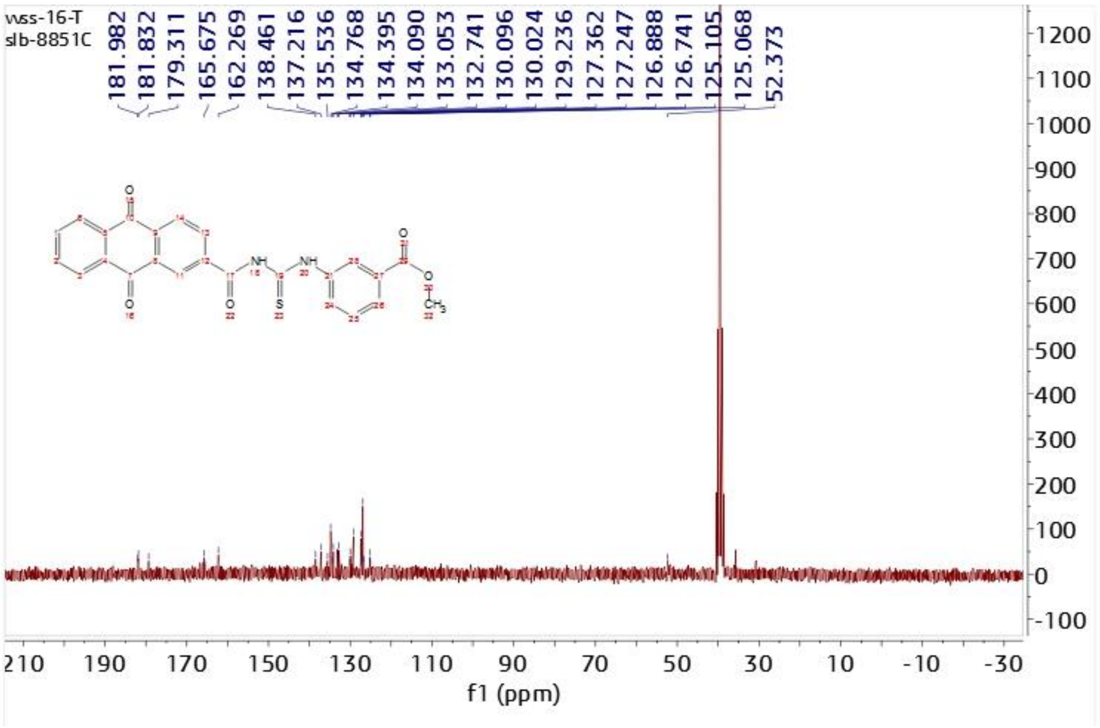
wss-16-T
slb-8851C
181.982 181.832 179.311 165.675 162.269 138.461 137.216 135.536 134.768 134.395 134.090 133.053 132.741 130.096 130.024 129.236 127.362 127.247 126.888 126.741 125.105 125.068 52.373
f1 (ppm)

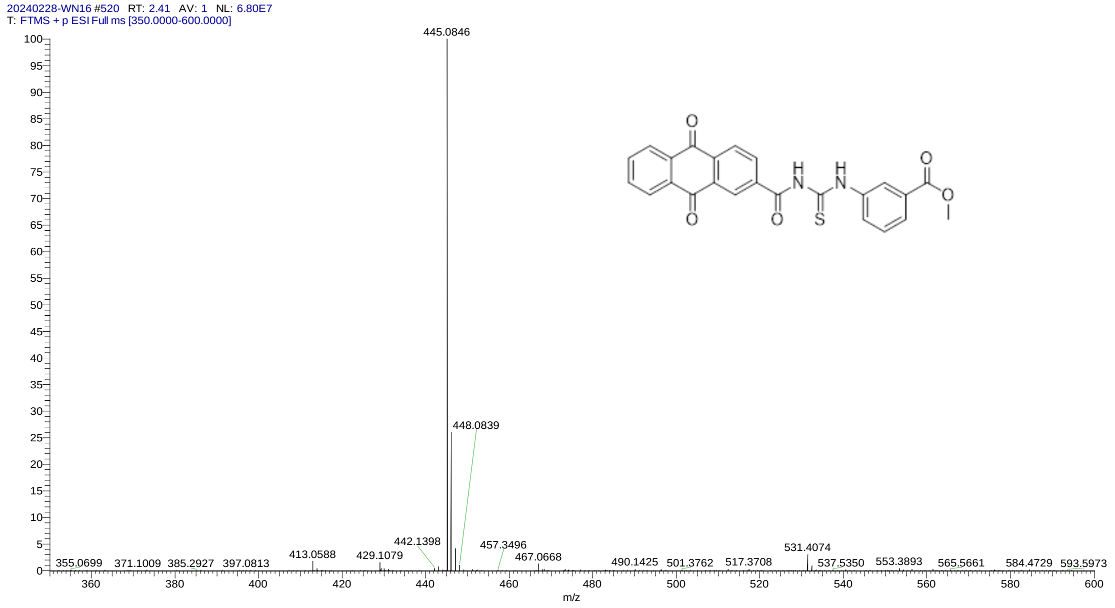

Data S8. ^{1}H-NMR, ^{13}C-NMR and HRMS data of compound 5h

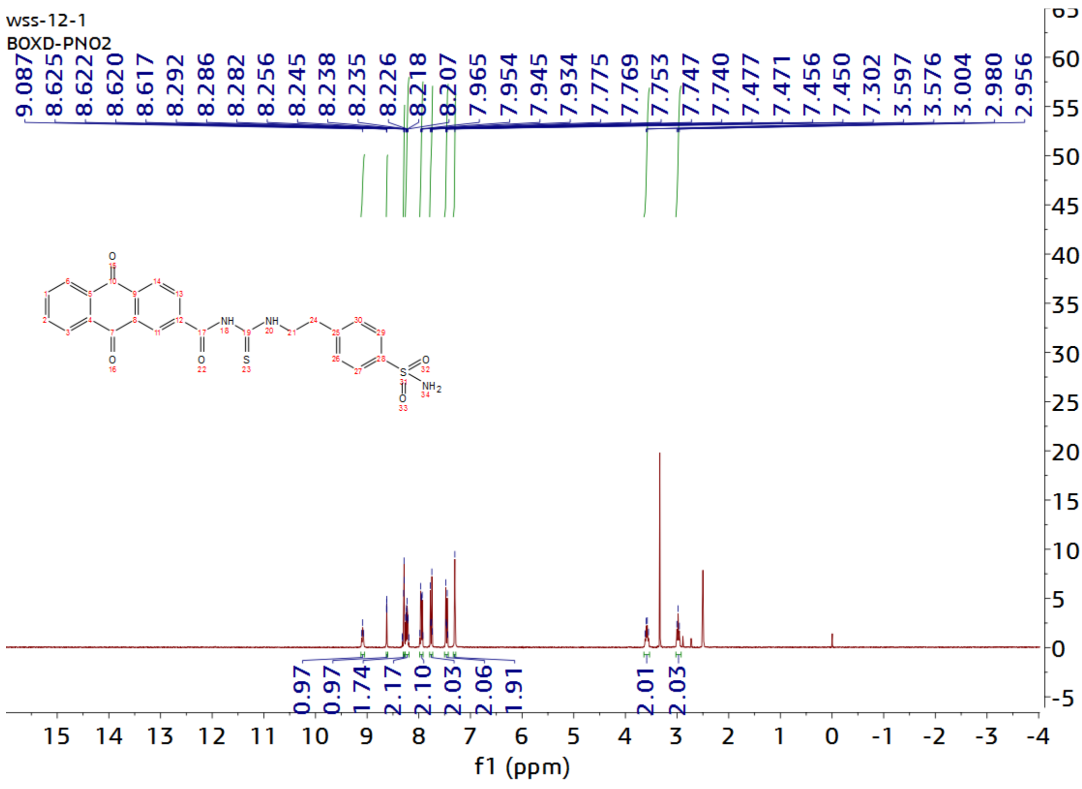

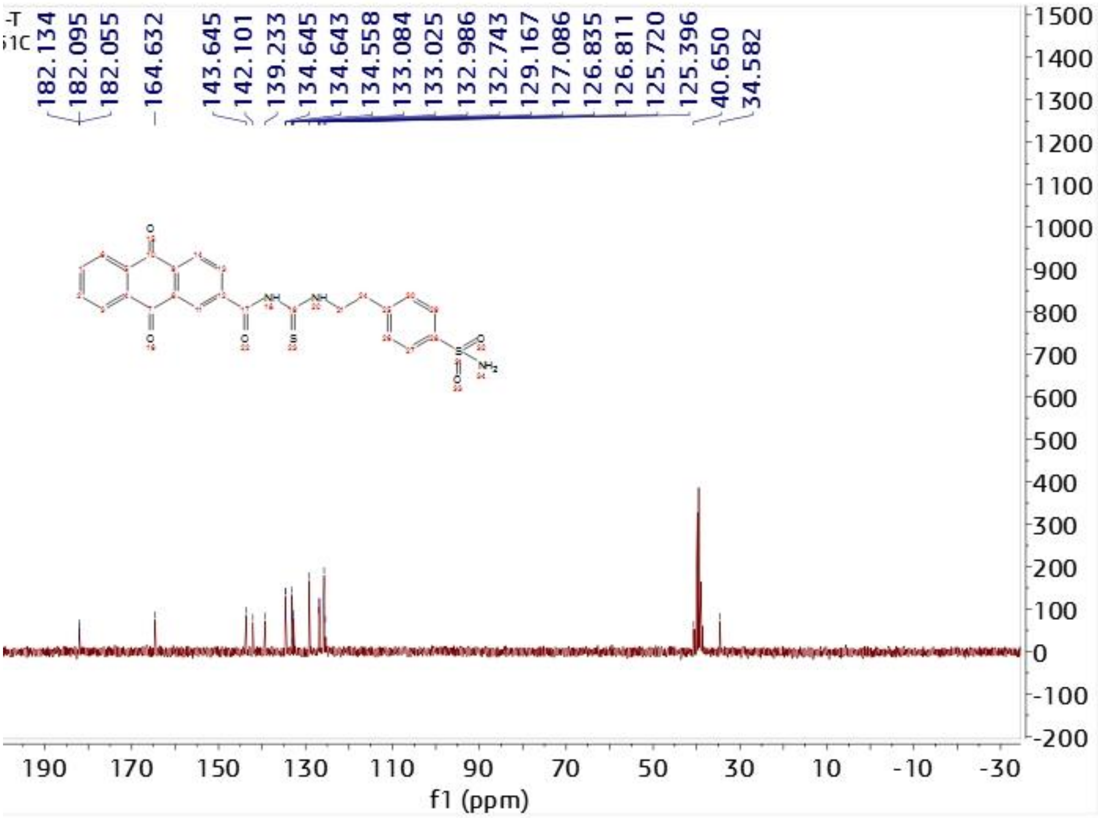

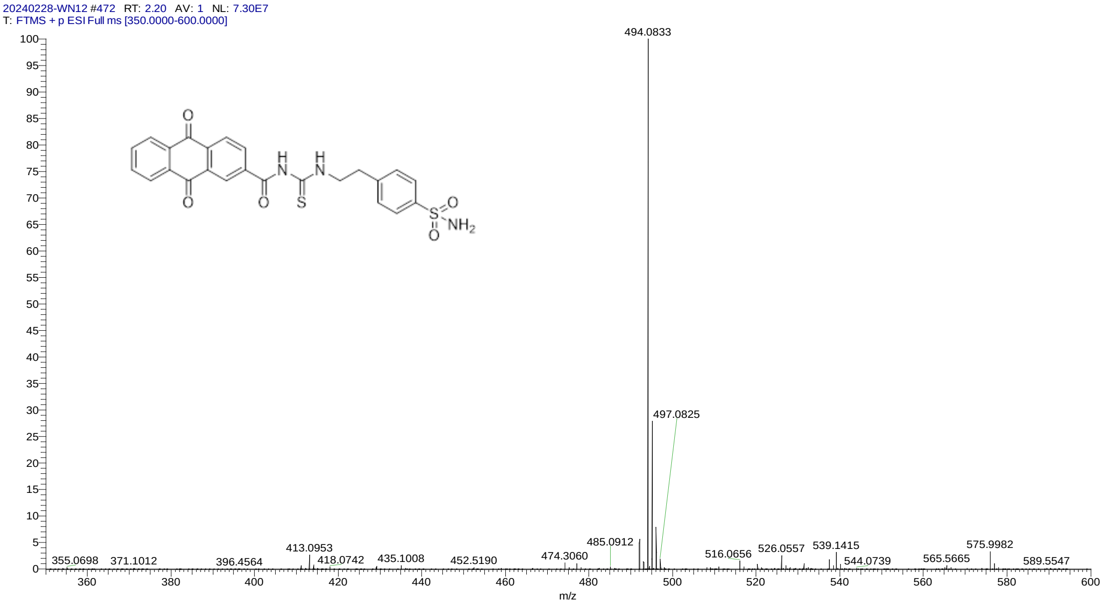

Data S9. ^{1}H-NMR, ^{13}C-NMR and HRMS data of compound 5i

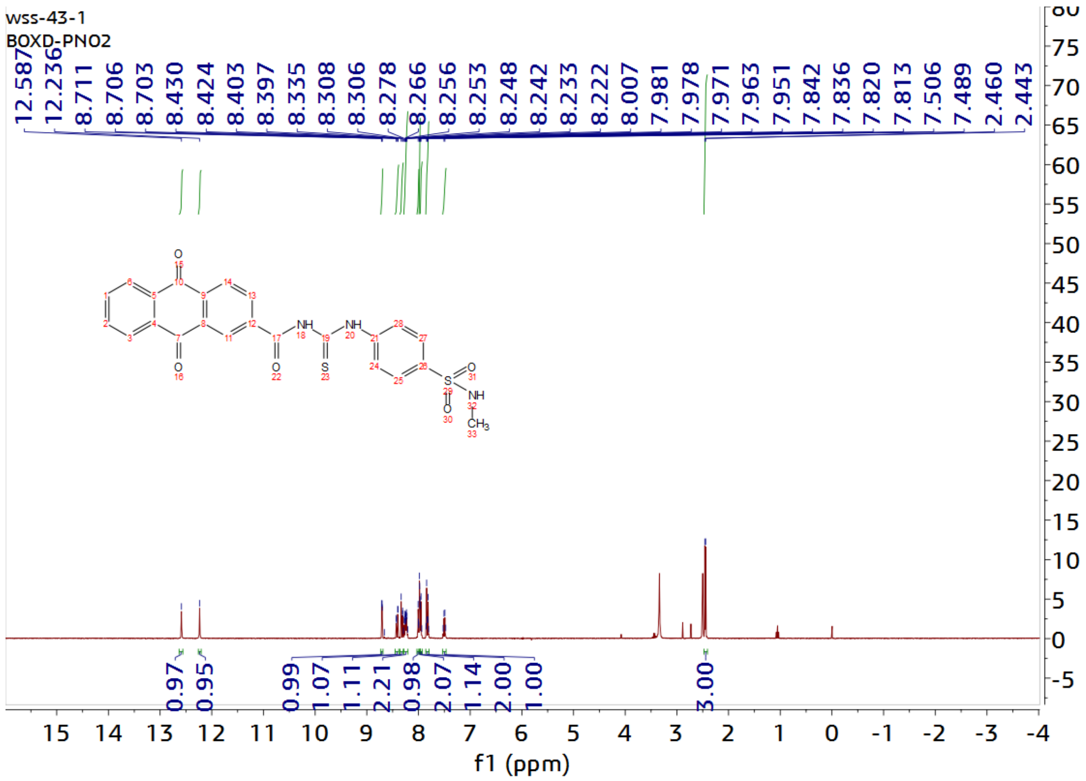
wss-43-1
BOXD-PNO2
12.587
12.236
8.711
8.706
8.703
8.430
8.424
8.403
8.397
8.335
8.308
8.306
8.278
8.266
8.256
8.253
8.248
8.242
8.233
8.222
8.007
7.981
7.978
7.971
7.963
7.951
7.842
7.836
7.820
7.813
7.506
7.489
2.460
2.443
0.97
0.95
0.99
1.07
1.11
2.21
0.98
2.07
1.14
2.00
1.00
3.00
f1 (ppm)

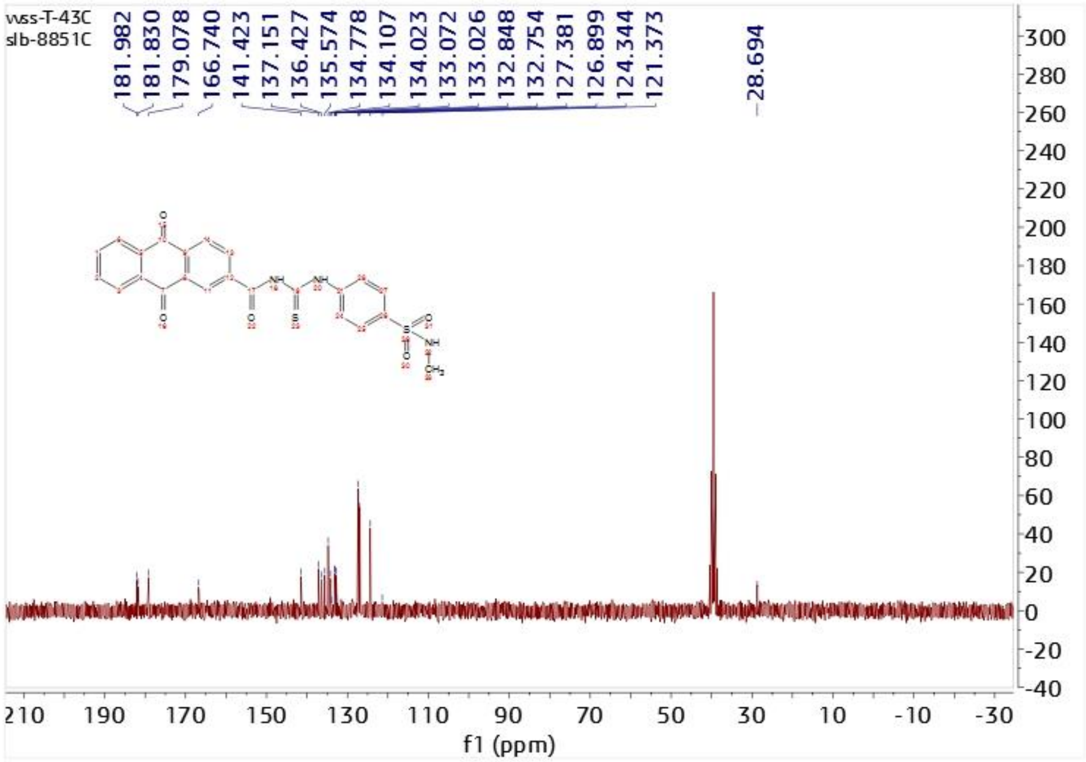
wss-T-43C
slb-8851C
181.982
181.830
179.078
166.740
141.423
137.151
136.427
135.574
134.778
134.107
134.023
133.072
133.026
132.848
132.754
127.381
126.899
124.344
121.373
28.694
f1 (ppm)

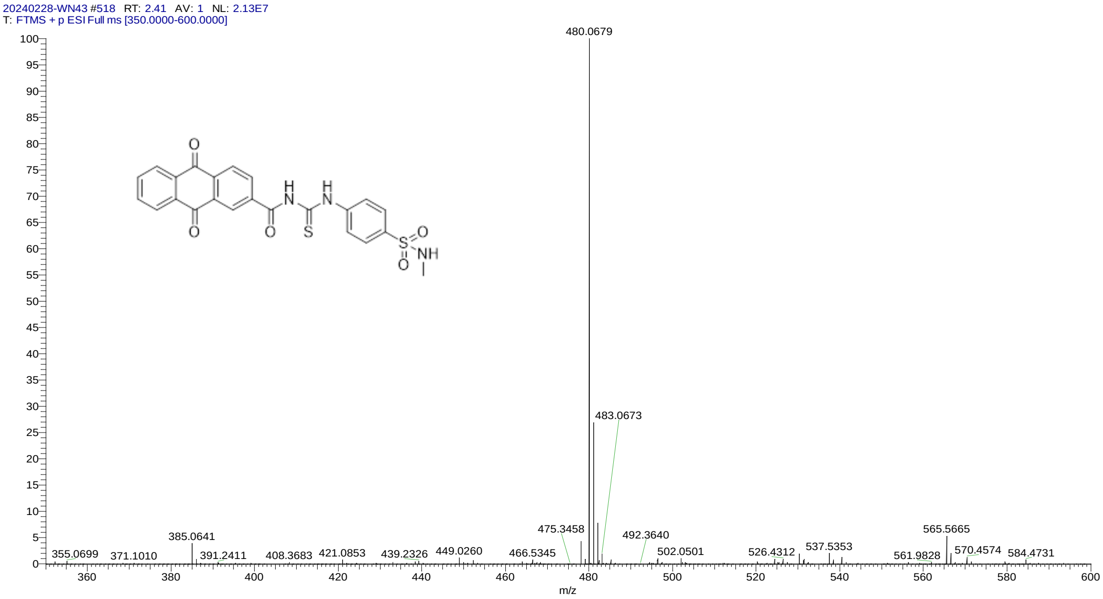

Data S10. ^{1}H-NMR, ^{13}C-NMR and HRMS data of compound 5j

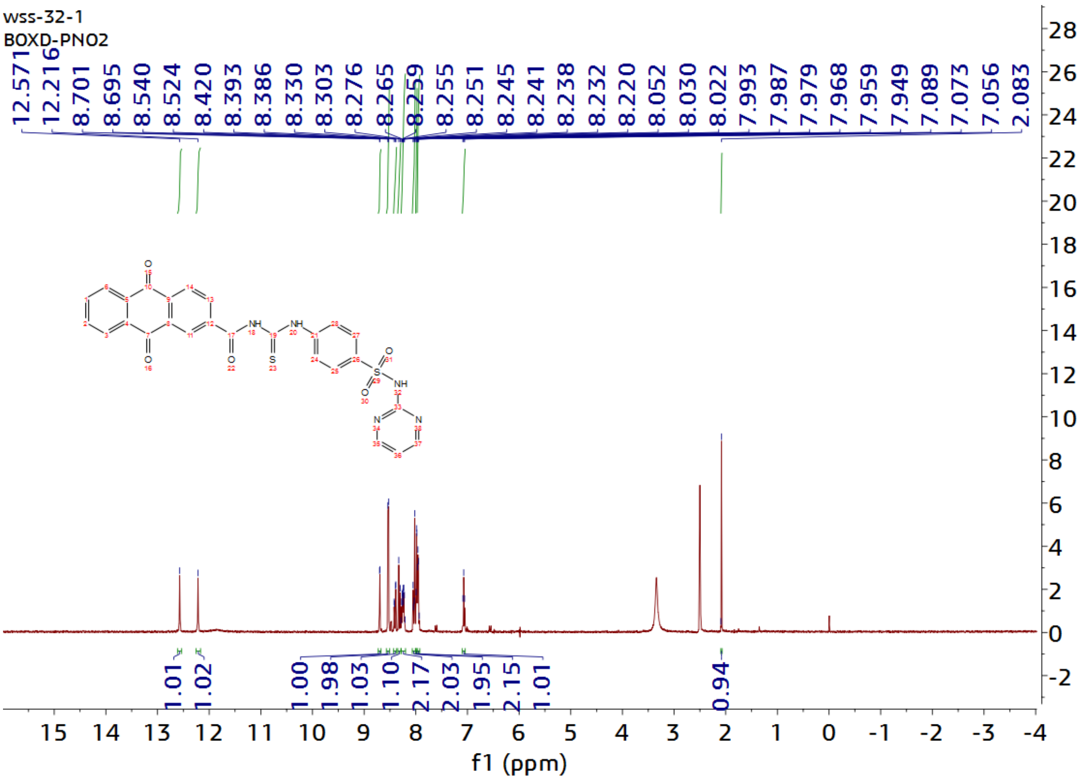

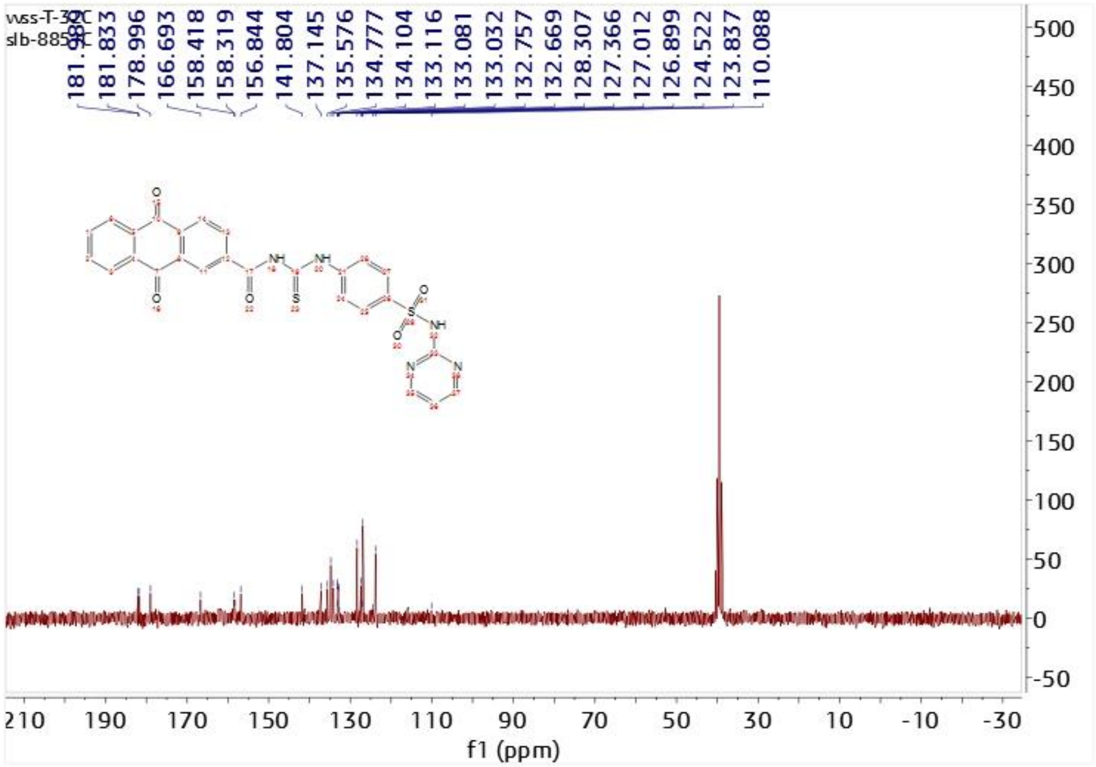

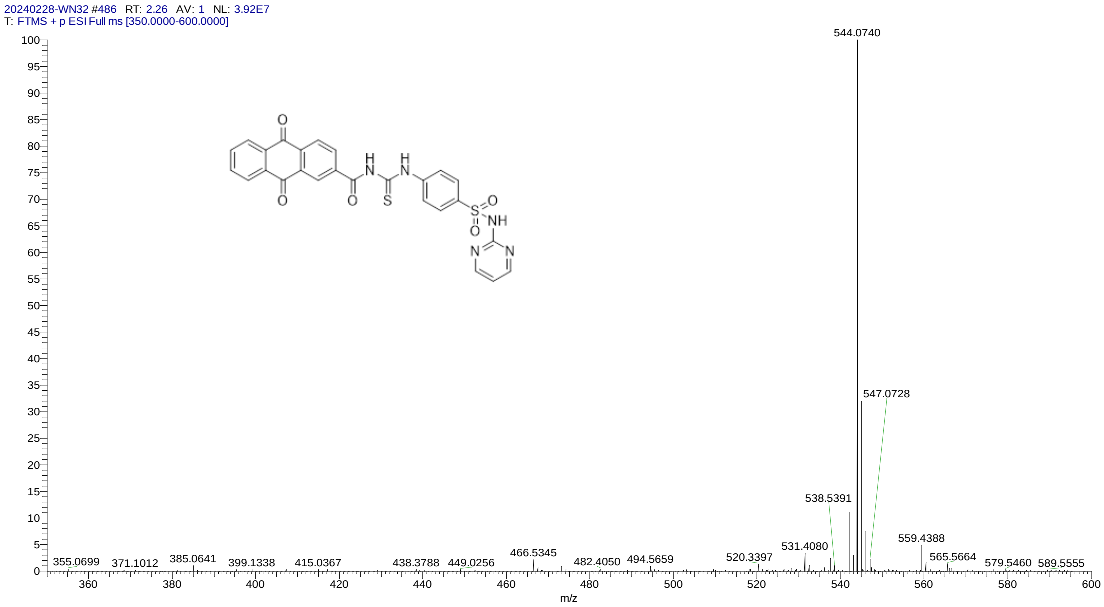

Data S11. ^{1}H-NMR, ^{13}C-NMR and HRMS data of compound 5k

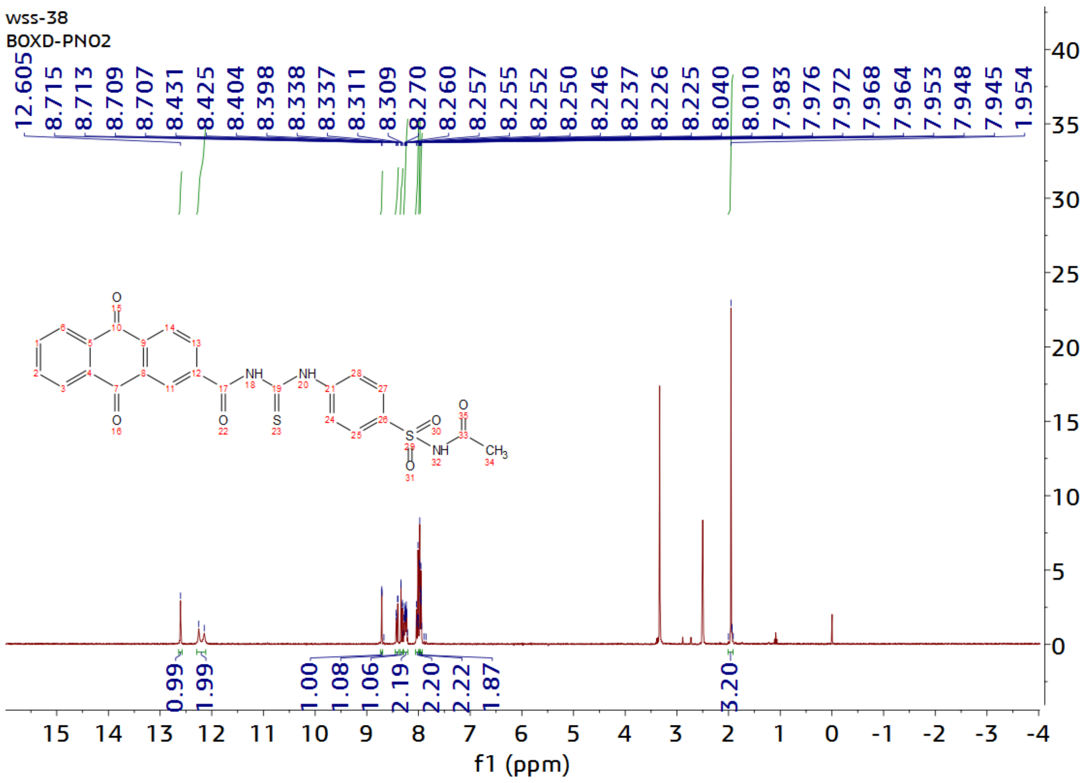
wss-38
BOXD-PNO2
12.605
8.715
8.713
8.709
8.707
8.431
8.425
8.404
8.398
8.338
8.337
8.311
8.309
8.270
8.260
8.257
8.255
8.252
8.250
8.246
8.237
8.226
8.225
8.040
8.010
7.983
7.976
7.972
7.968
7.964
7.953
7.948
7.945
1.954
0.99
1.99
1.00
1.08
1.06
2.19
2.20
2.22
1.87
3.20
f1 (ppm)

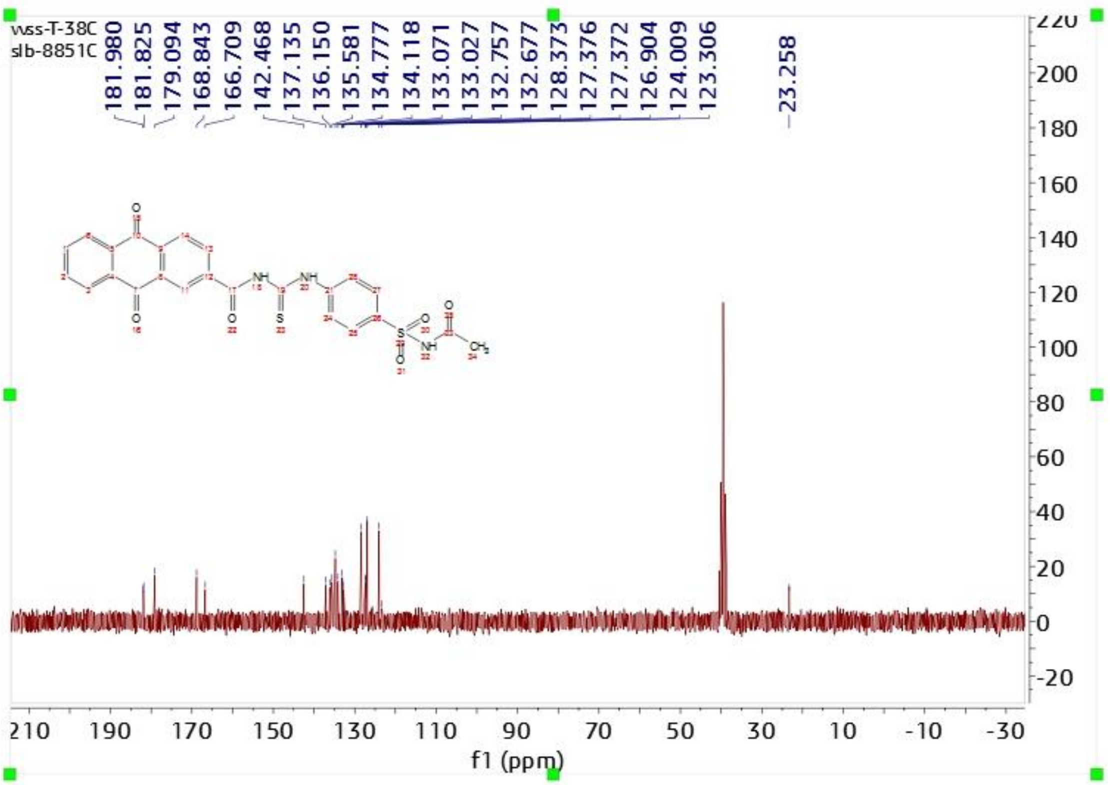
wss-T-38C
slb-8851C
181.980
181.825
179.094
168.843
166.709
142.468
137.135
136.150
135.581
134.777
134.118
133.071
133.027
132.757
132.677
128.373
127.376
127.372
126.904
124.009
123.306
23.258
f1 (ppm)

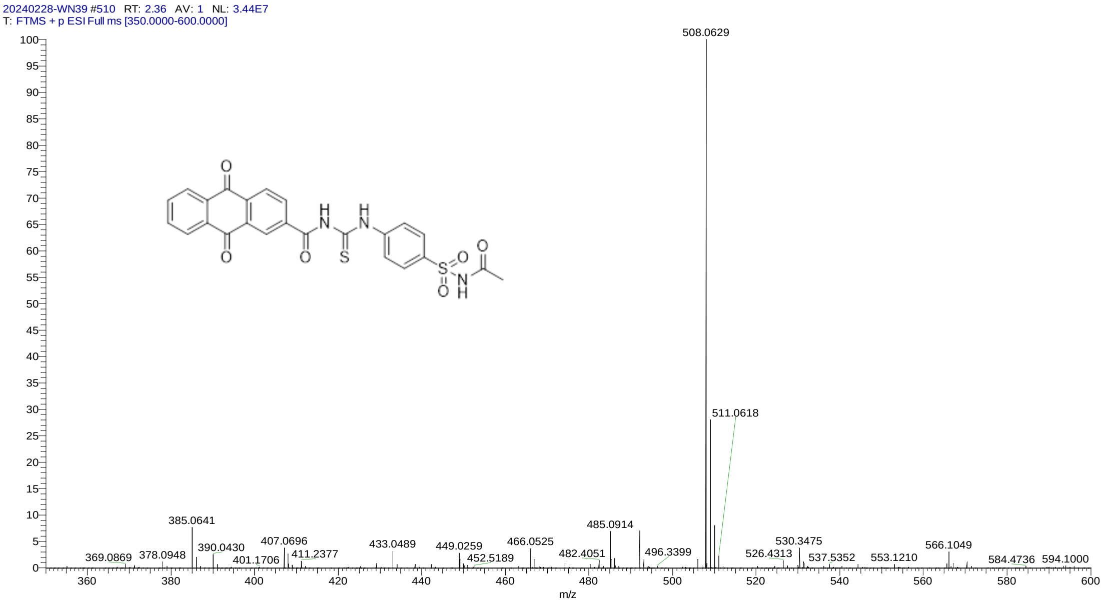

Data S12. ^{1}H-NMR, ^{13}C-NMR and HRMS data of compound 5l

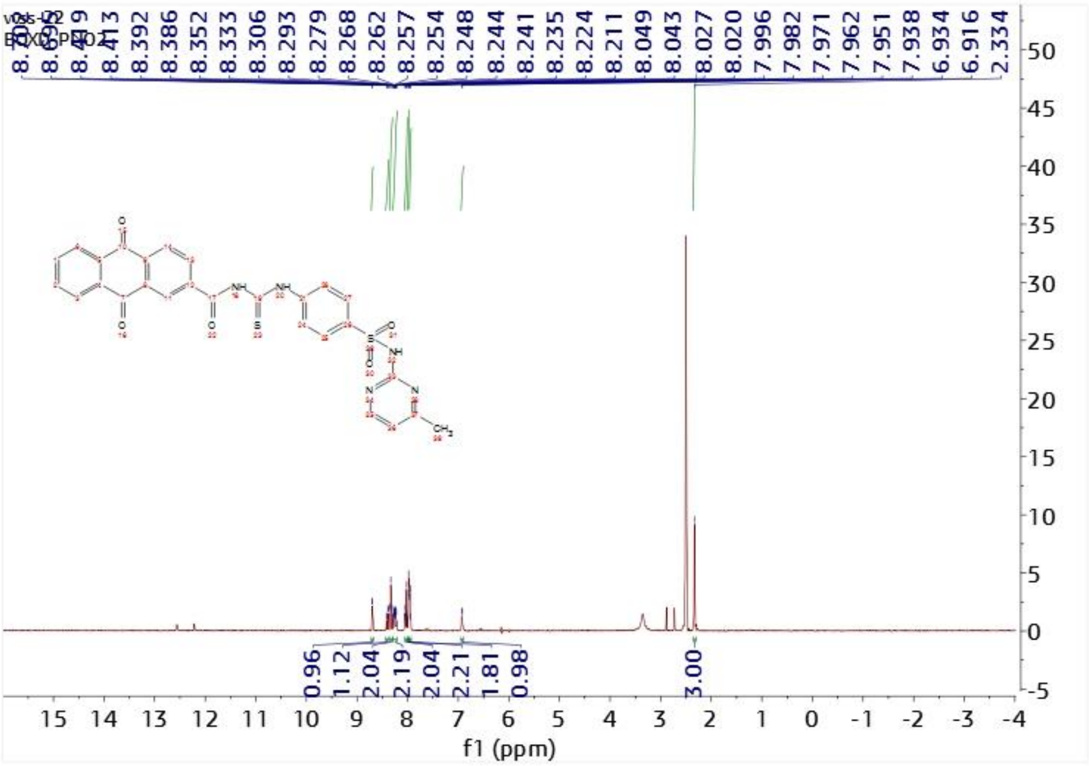

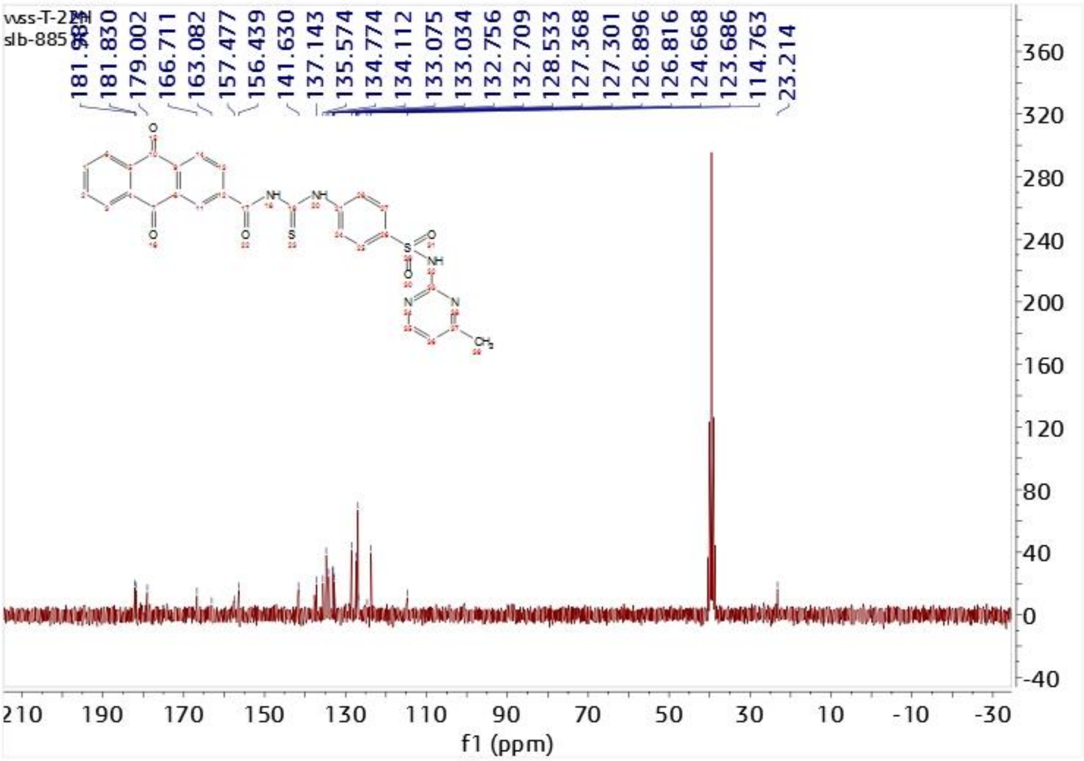

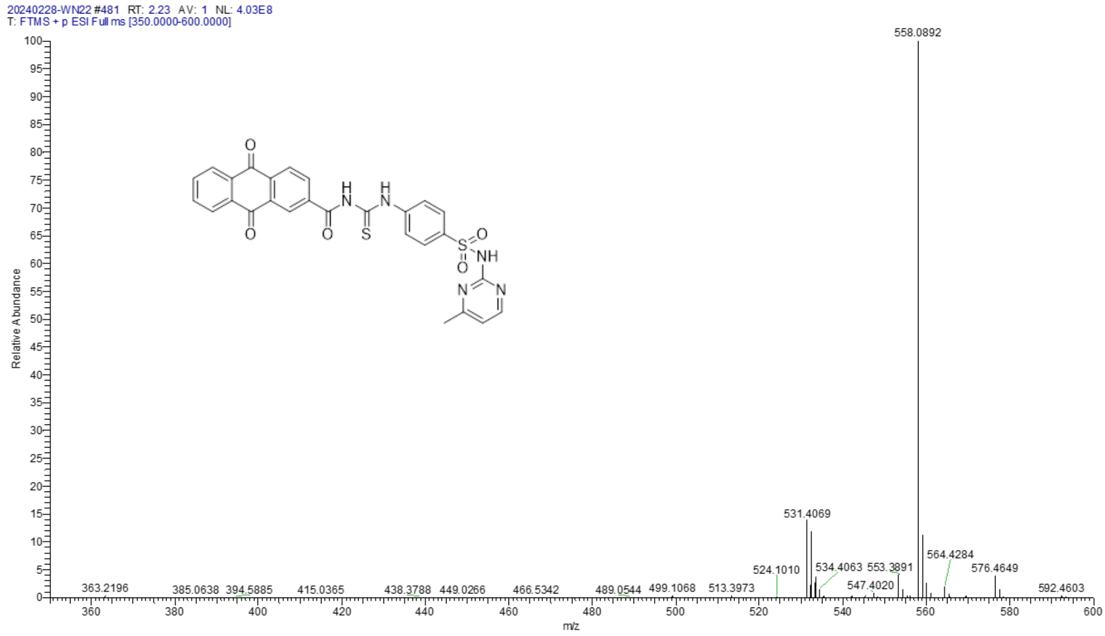

Data S13. ^{1}H-NMR, ^{13}C-NMR and HRMS data of compound 5m

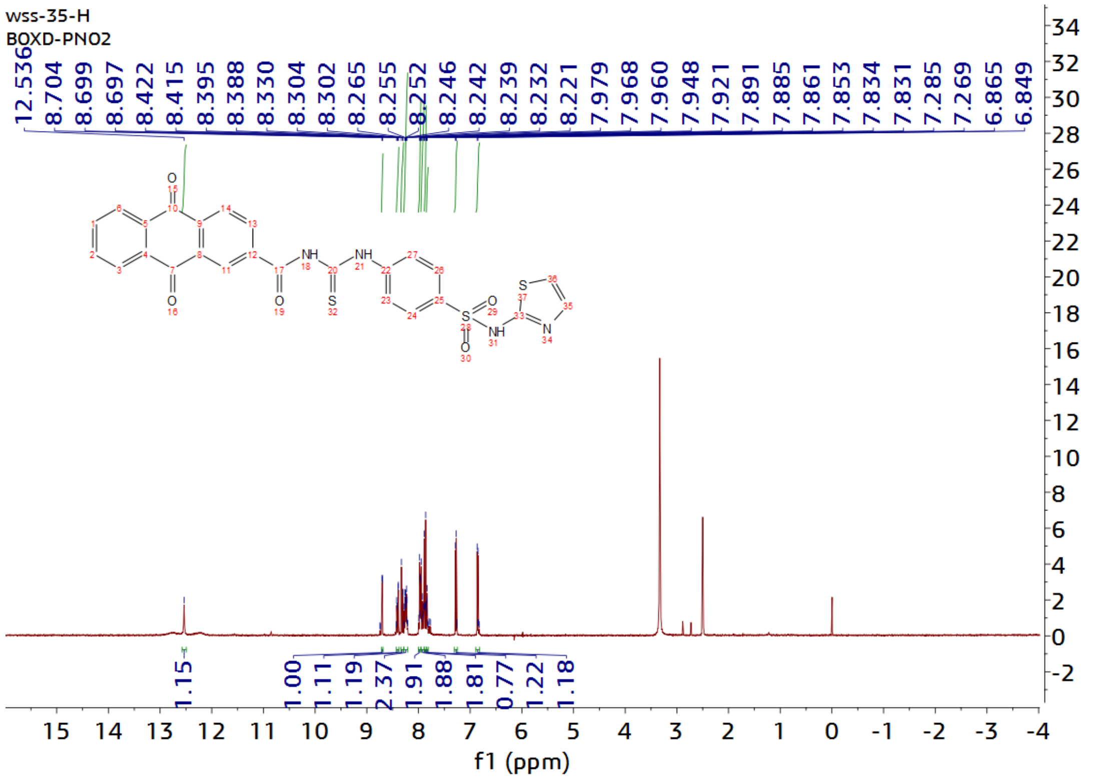
wss-35-H
BOXD-PNO2
12.536
8.704
8.699
8.697
8.422
8.415
8.395
8.388
8.330
8.304
8.302
8.265
8.255
8.252
8.246
8.242
8.239
8.232
8.221
7.979
7.968
7.960
7.948
7.921
7.891
7.885
7.861
7.853
7.834
7.831
7.285
7.269
6.865
6.849
1.15
1.00
1.11
1.19
2.37
1.91
1.88
1.81
0.77
1.22
1.18
f1 (ppm)

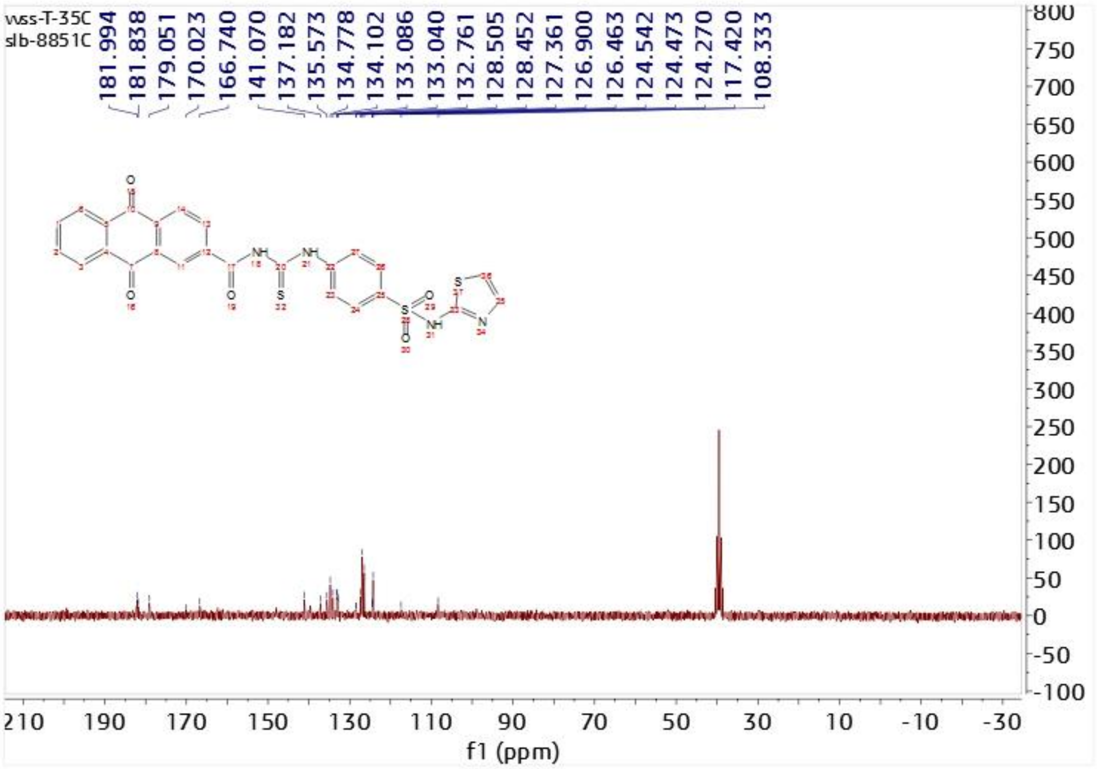
wss-T-35C
slb-8851C
181.994
181.838
179.051
170.023
166.740
141.070
137.182
135.573
134.778
134.102
133.086
133.040
132.761
128.505
128.452
127.361
126.900
126.463
124.542
124.473
124.270
117.420
108.333
f1 (ppm)

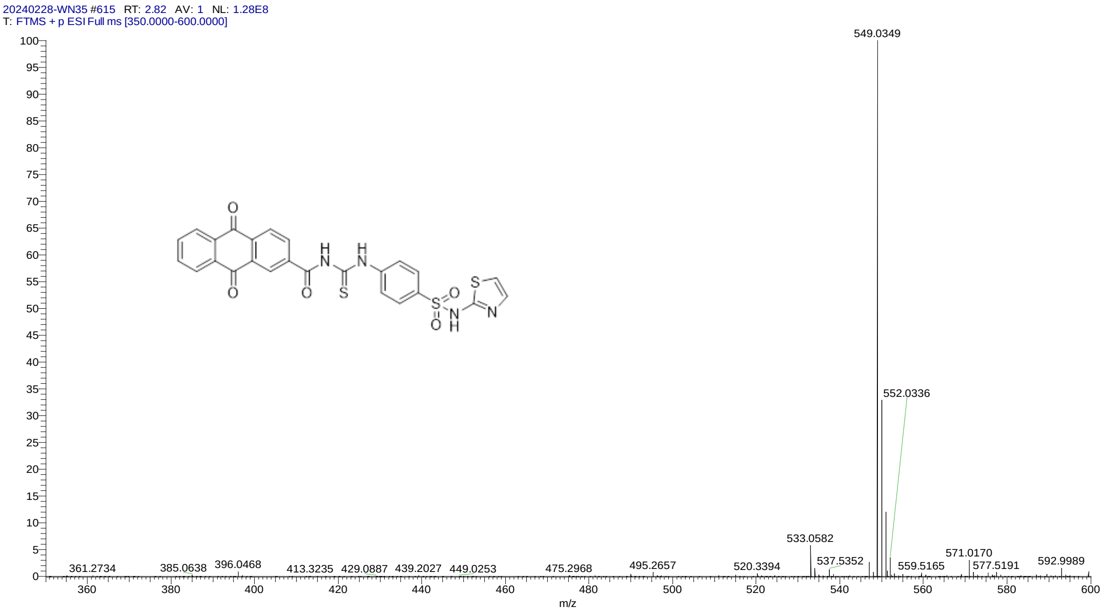

Data S14. ^{1}H-NMR, ^{13}C-NMR and HRMS data of compound 5n

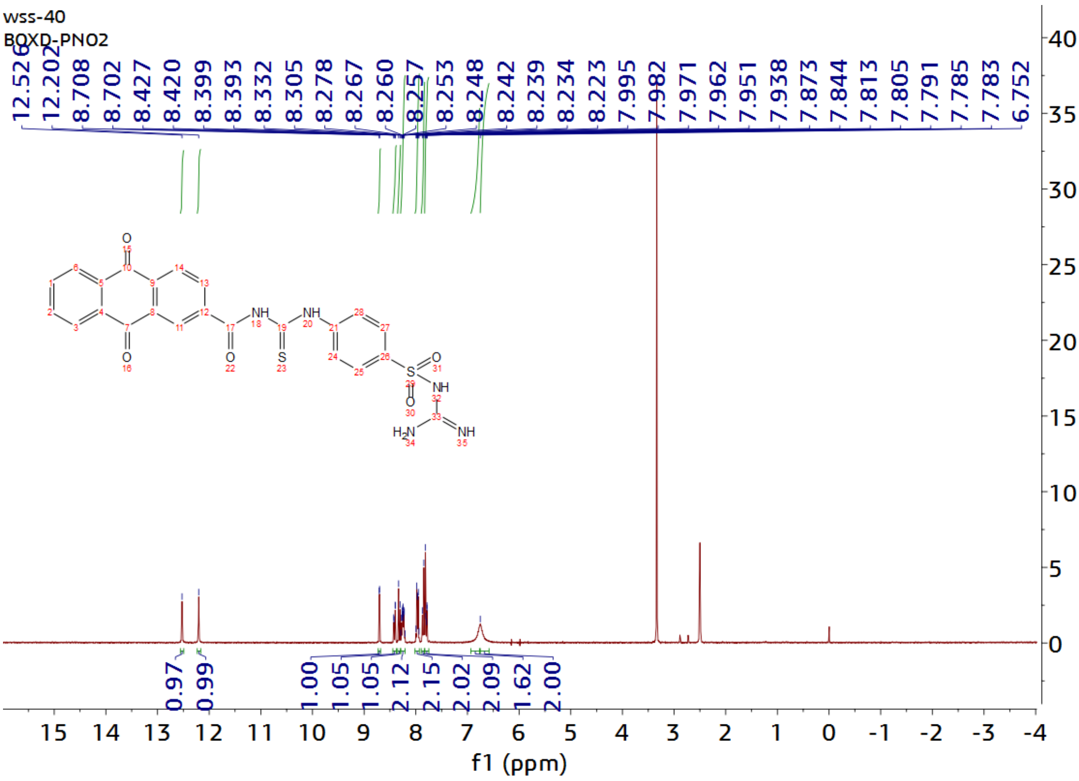

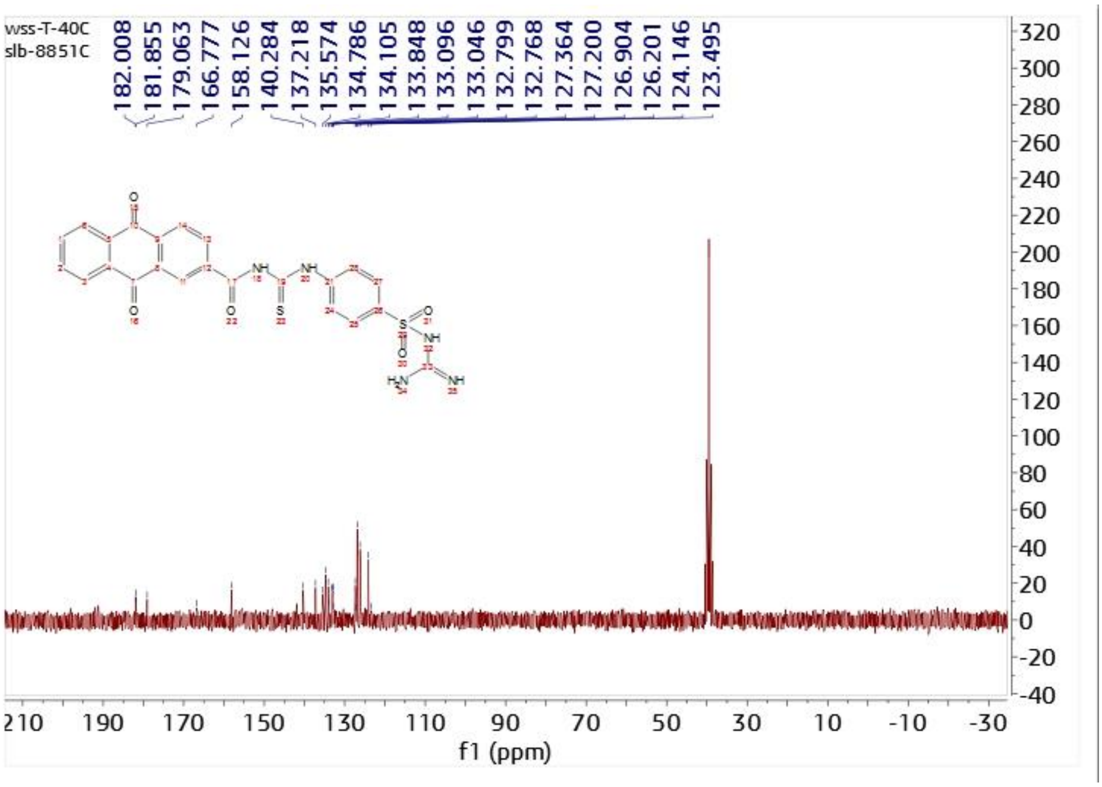

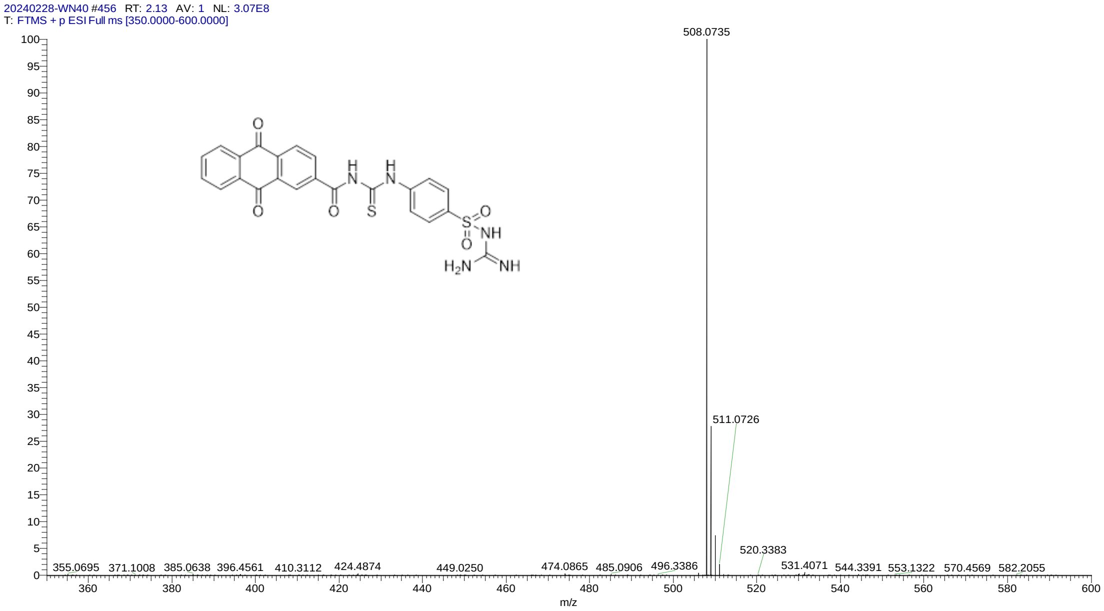

Data S15. ^{1}H-NMR, ^{13}C-NMR and HRMS data of compound 6a

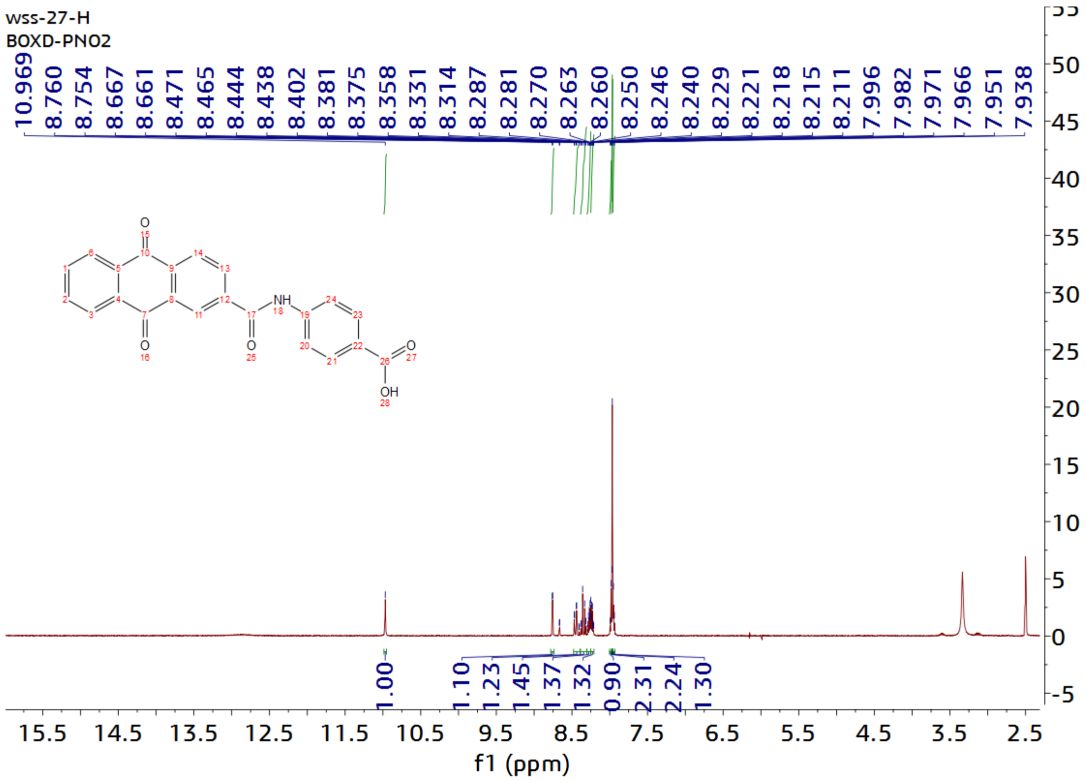
wss-27-H
BOXD-PNO2
10.969
8.760
8.754
8.667
8.661
8.471
8.465
8.444
8.438
8.402
8.381
8.375
8.358
8.331
8.314
8.287
8.281
8.270
8.263
8.260
8.250
8.246
8.240
8.229
8.221
8.218
8.215
8.211
7.996
7.982
7.971
7.966
7.951
7.938
NH
OH
1.00
1.10
1.23
1.45
1.37
1.32
0.90
2.31
2.24
1.30
f1 (ppm)

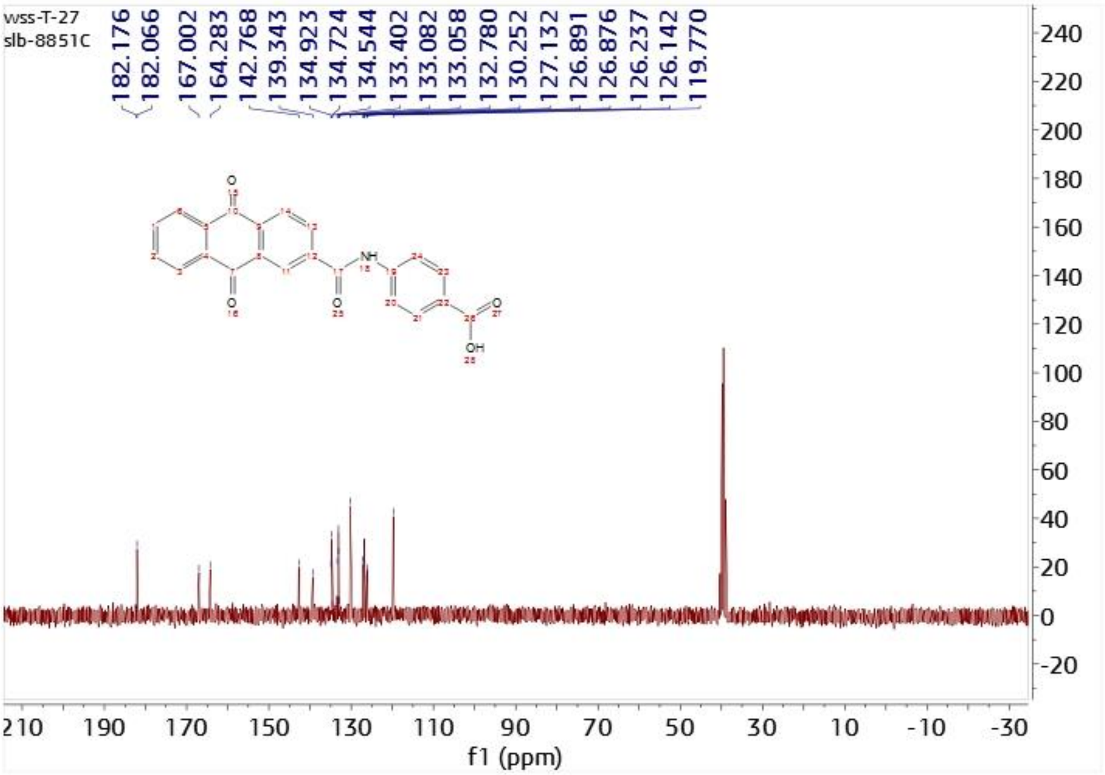
wss-T-27
slb-8851C
182.176
182.066
167.002
164.283
142.768
139.343
134.923
134.724
134.544
133.402
133.082
133.058
132.780
130.252
127.132
126.891
126.876
126.237
126.142
119.770
NH
OH
f1 (ppm)

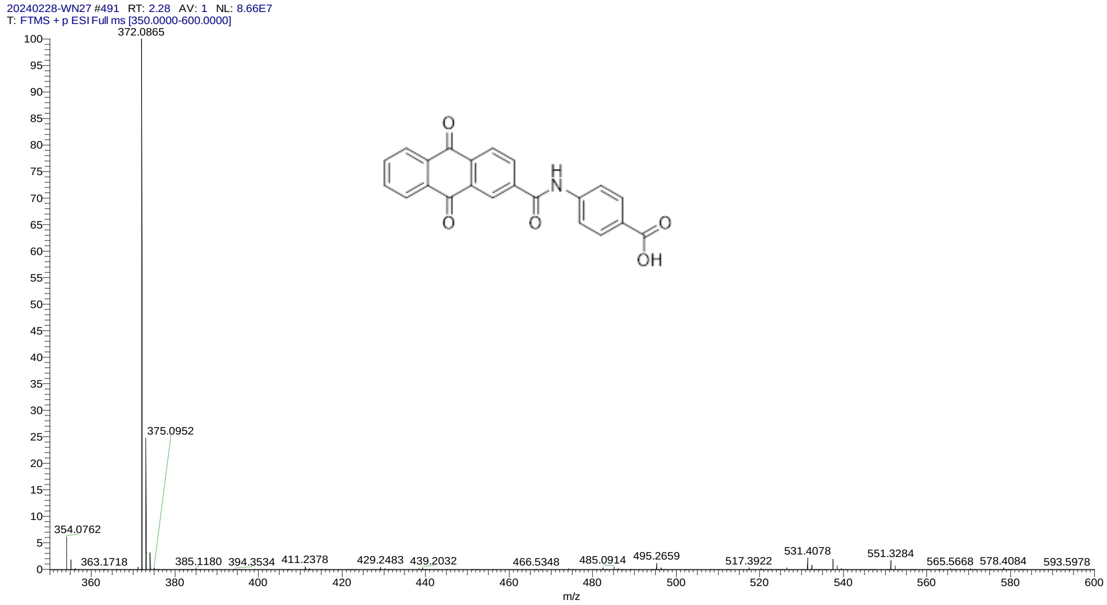

Data S16. ^{1}H-NMR, ^{13}C-NMR and HRMS data of compound 6b

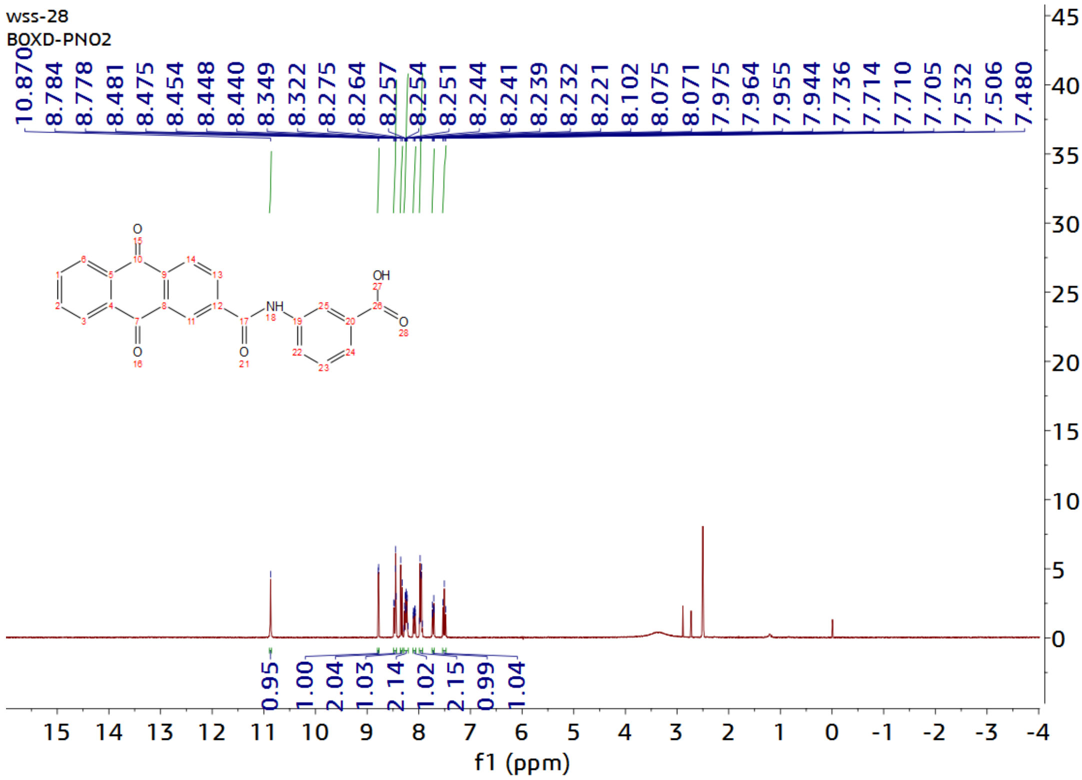

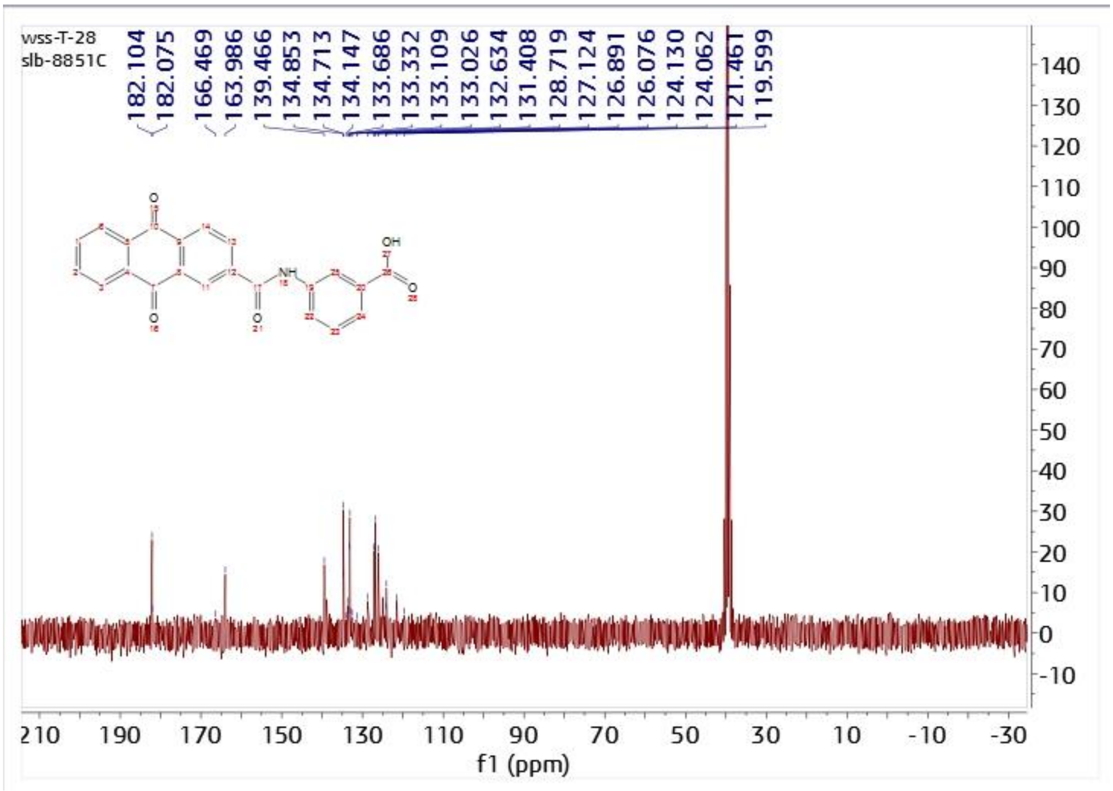

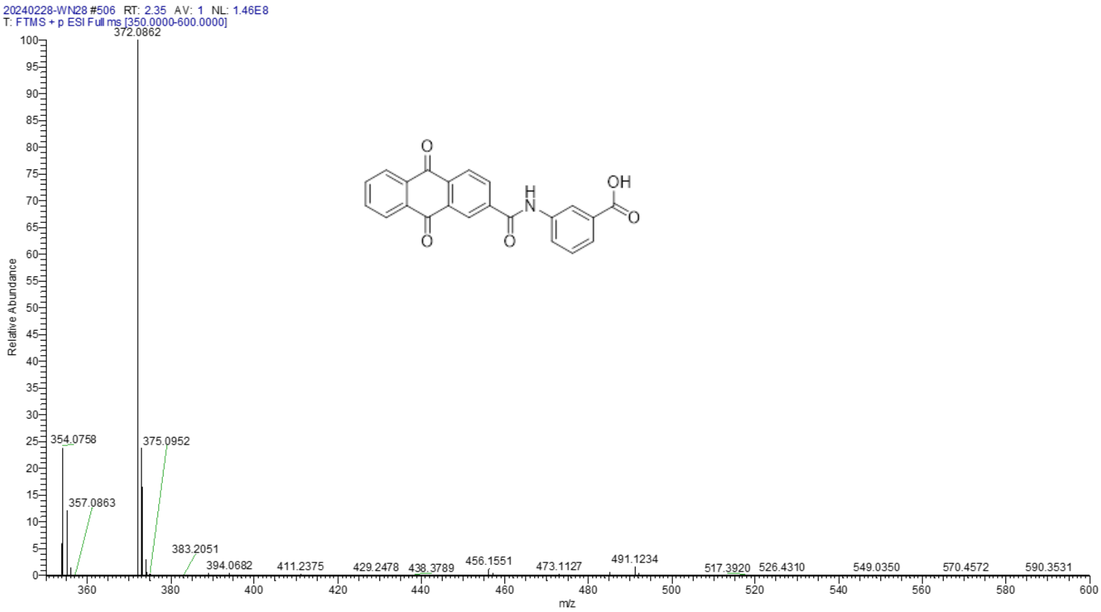

Data S17. ^{1}H-NMR, ^{13}C-NMR and HRMS data of compound 6c

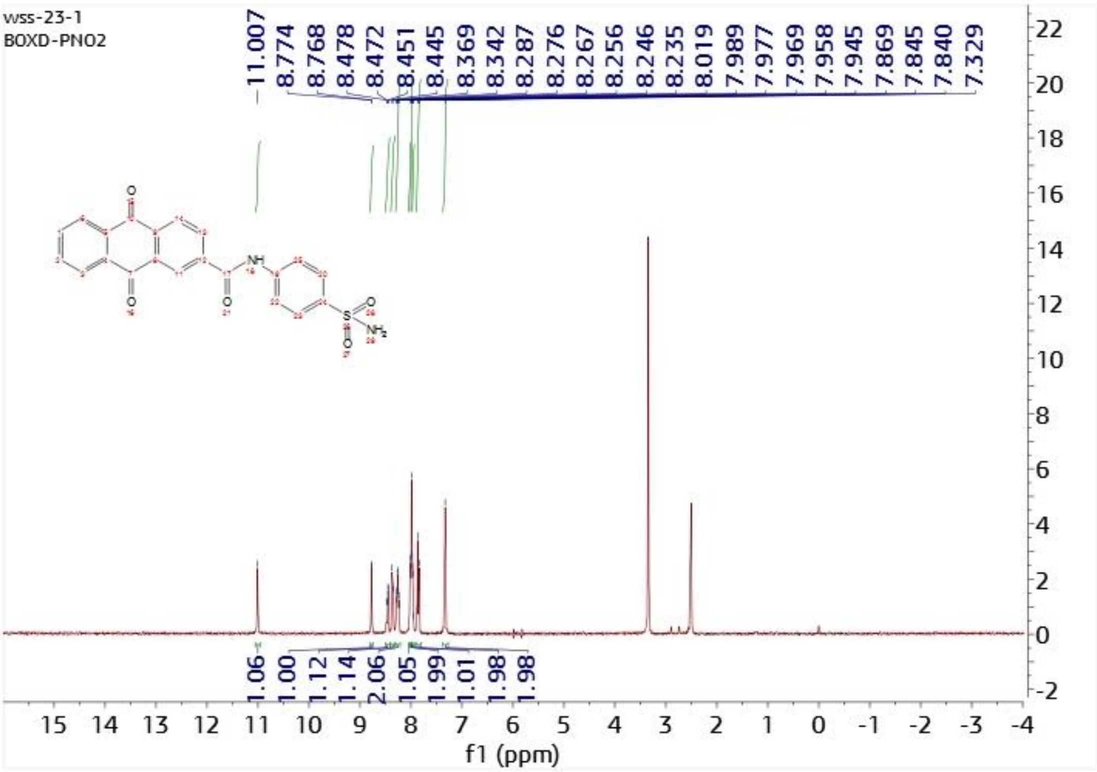
wss-23-1
BOXD-PNO2
11.007
8.774
8.768
8.478
8.472
8.451
8.445
8.369
8.342
8.287
8.276
8.267
8.256
8.246
8.235
8.019
7.989
7.977
7.969
7.958
7.945
7.869
7.845
7.840
7.329
1.06
1.00
1.12
1.14
2.06
1.05
1.99
1.01
1.98
1.98
f1 (ppm)

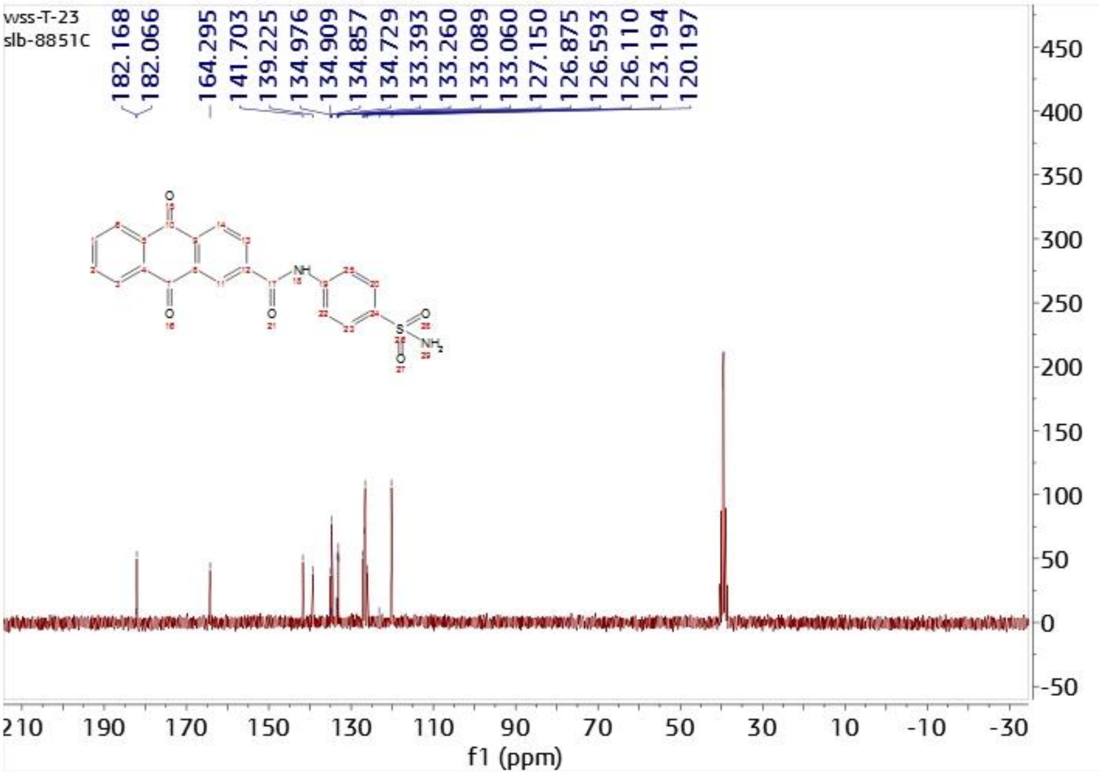
wss-T-23
slb-8851C
182.168
182.066
164.295
141.703
139.225
134.976
134.909
134.857
134.729
133.393
133.260
133.089
133.060
127.150
126.875
126.593
126.110
123.194
120.197
f1 (ppm)

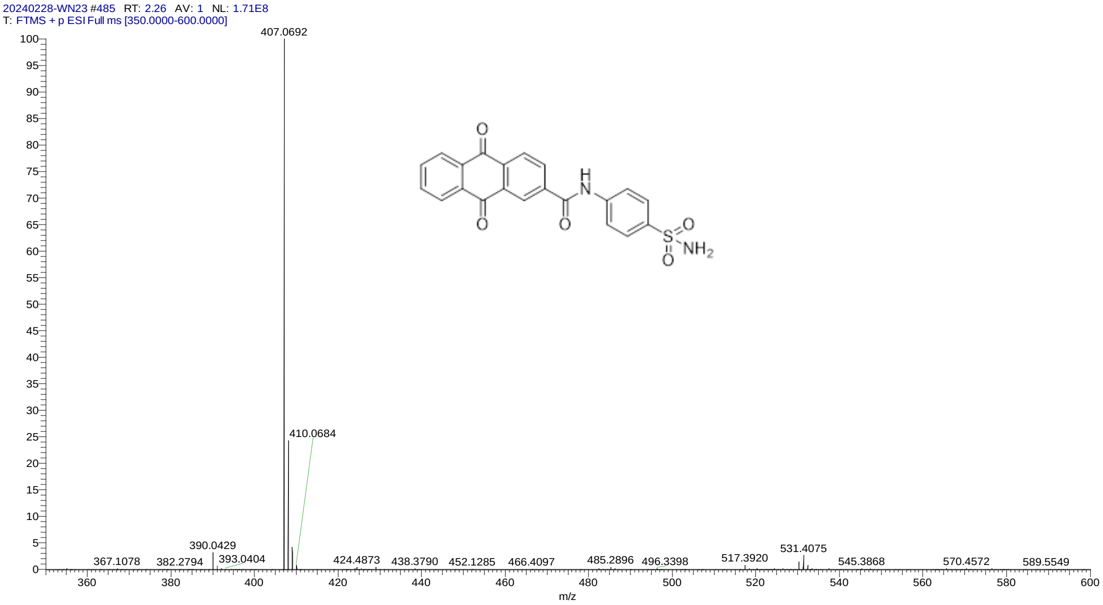

Data S18. ^{1}H-NMR, ^{13}C-NMR and HRMS data of compound 6d

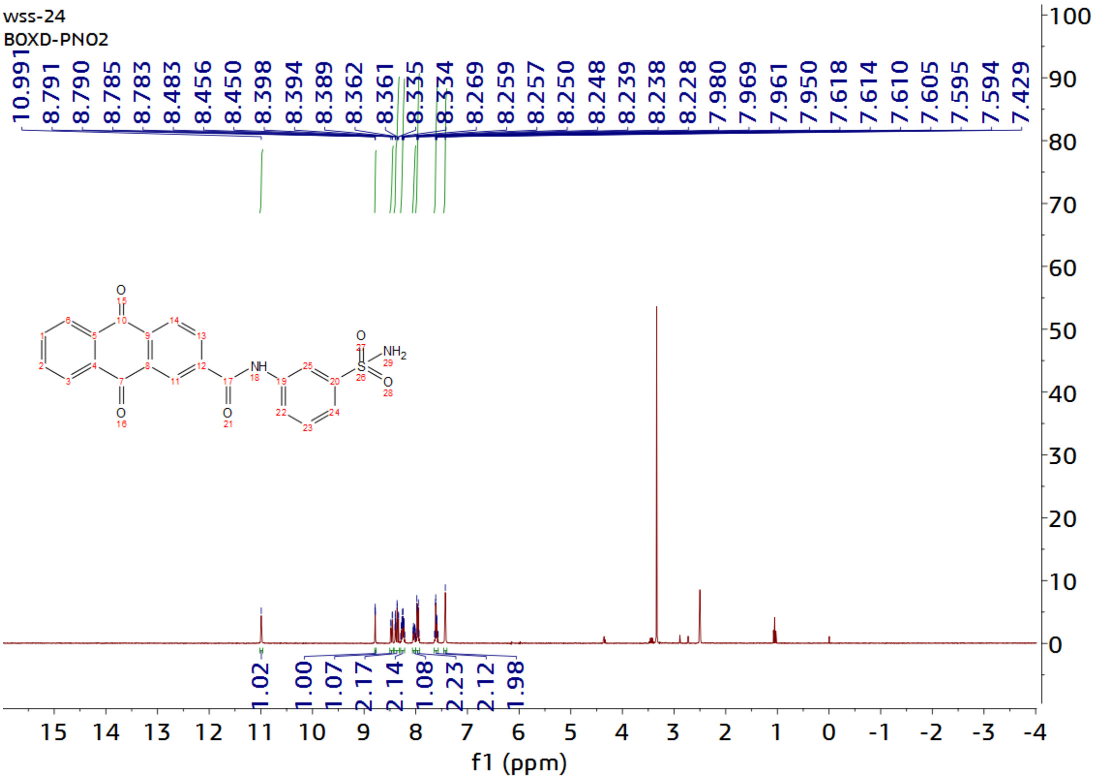

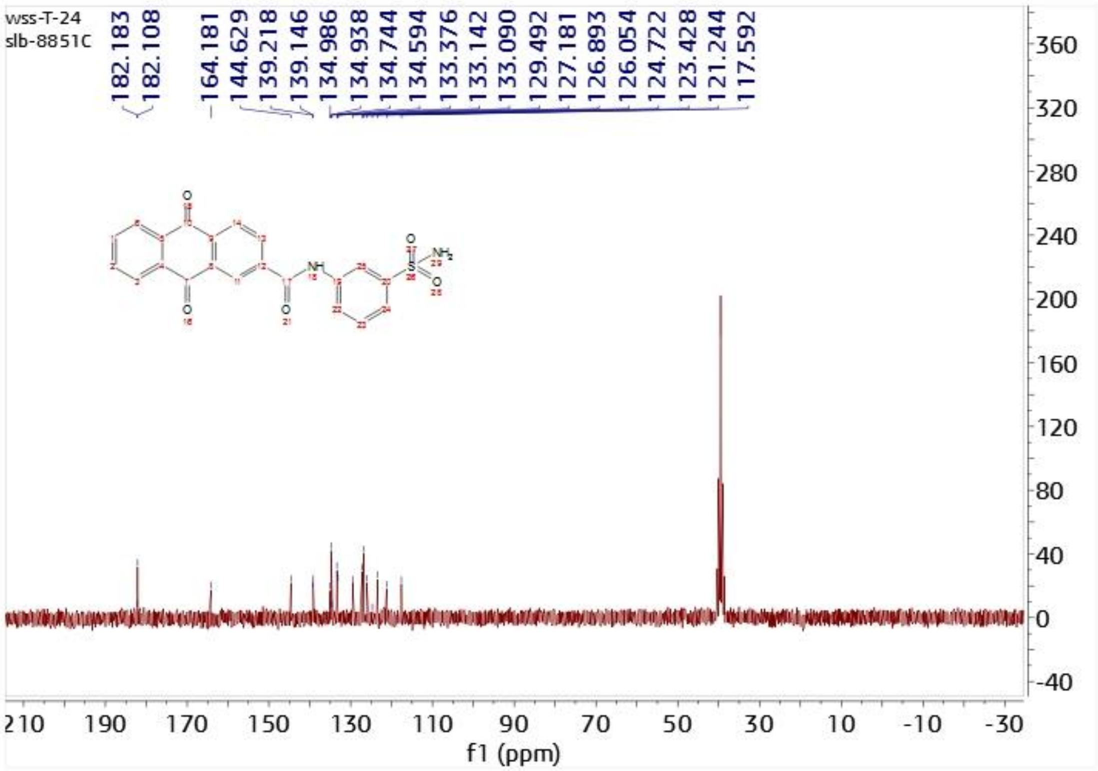

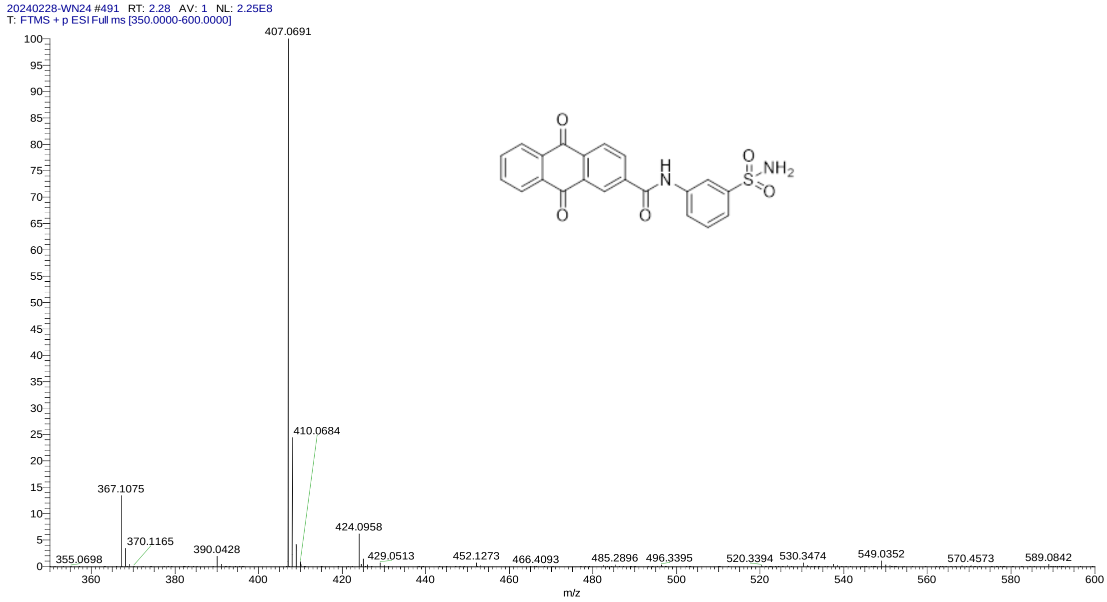

Data S19. ¹H-NMR, ¹³C-NMR and HRMS data of compound 6e

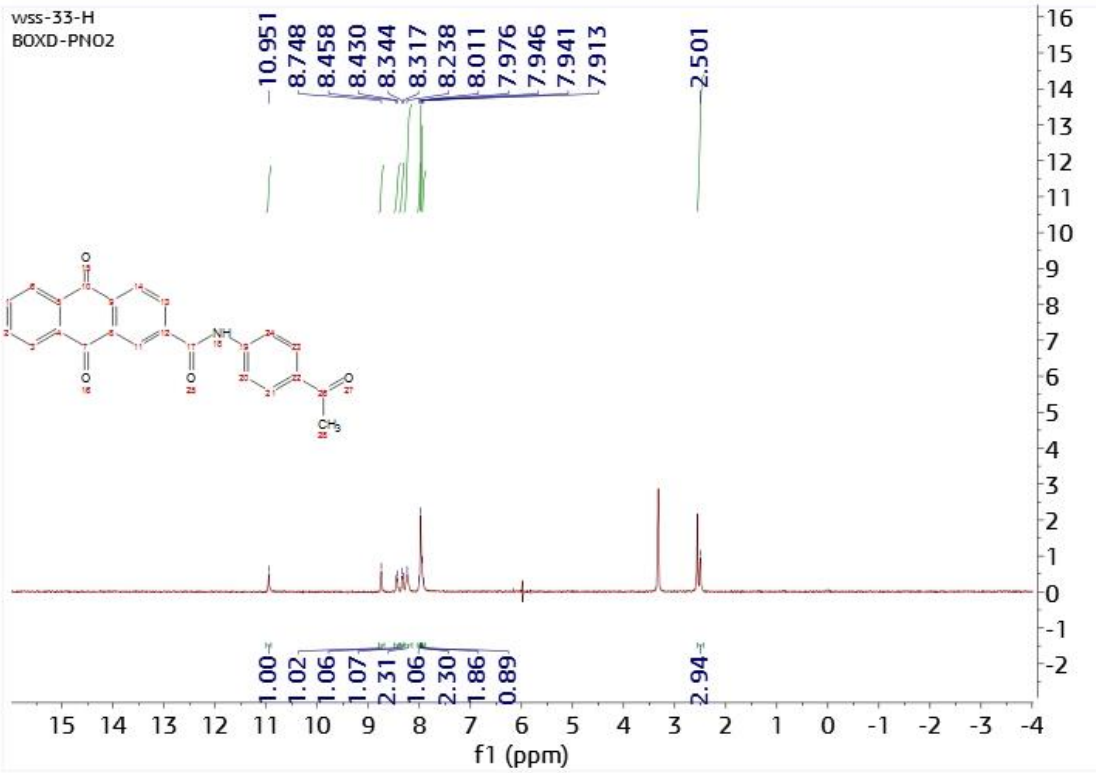

wss-33-H
BOXD-PNO2
10.951
8.748
8.458
8.430
8.344
8.317
8.238
8.011
7.976
7.946
7.941
7.913
2.501
NH
CH3
1.00
1.02
1.06
1.07
2.31
1.06
2.30
1.86
0.89
2.94
f1 (ppm)

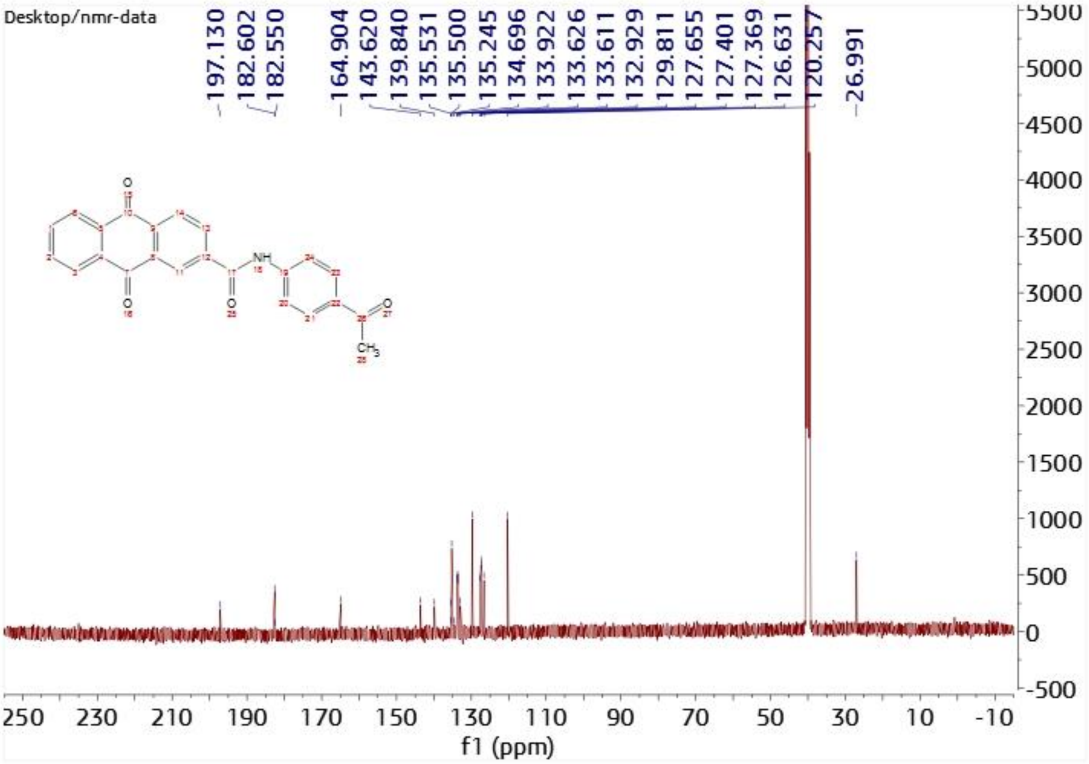

Desktop/nmr-data
197.130
182.602
182.550
164.904
143.620
139.840
135.531
135.500
135.245
134.696
133.922
133.626
133.611
132.929
129.811
127.655
127.401
127.369
126.631
120.257
26.991
NH
CH3
f1 (ppm)

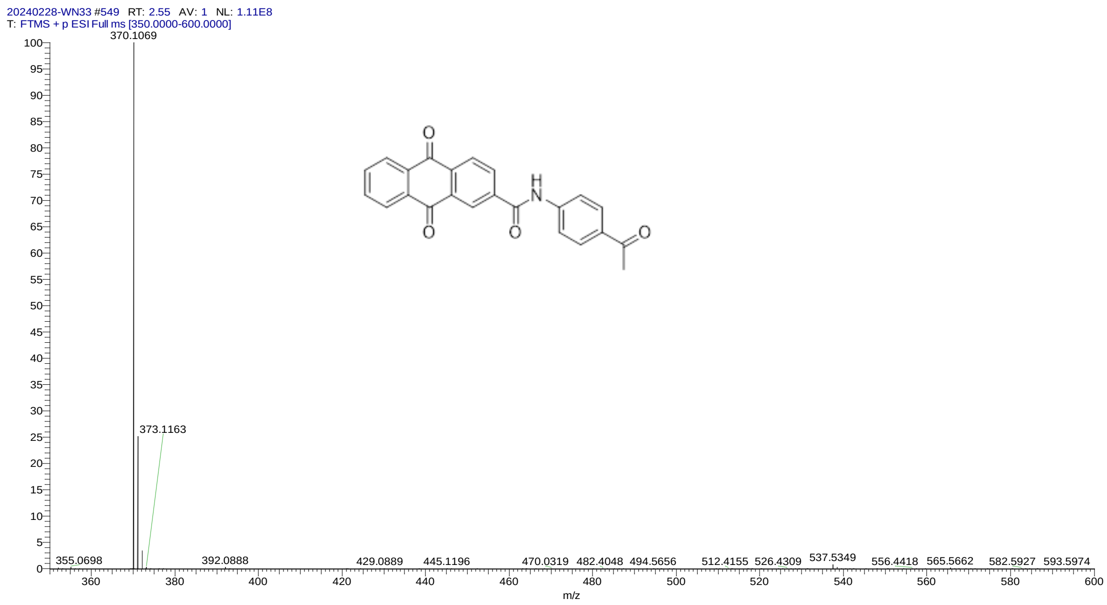

Data S20. ^{1}H-NMR, ^{13}C-NMR and HRMS data of compound 6h

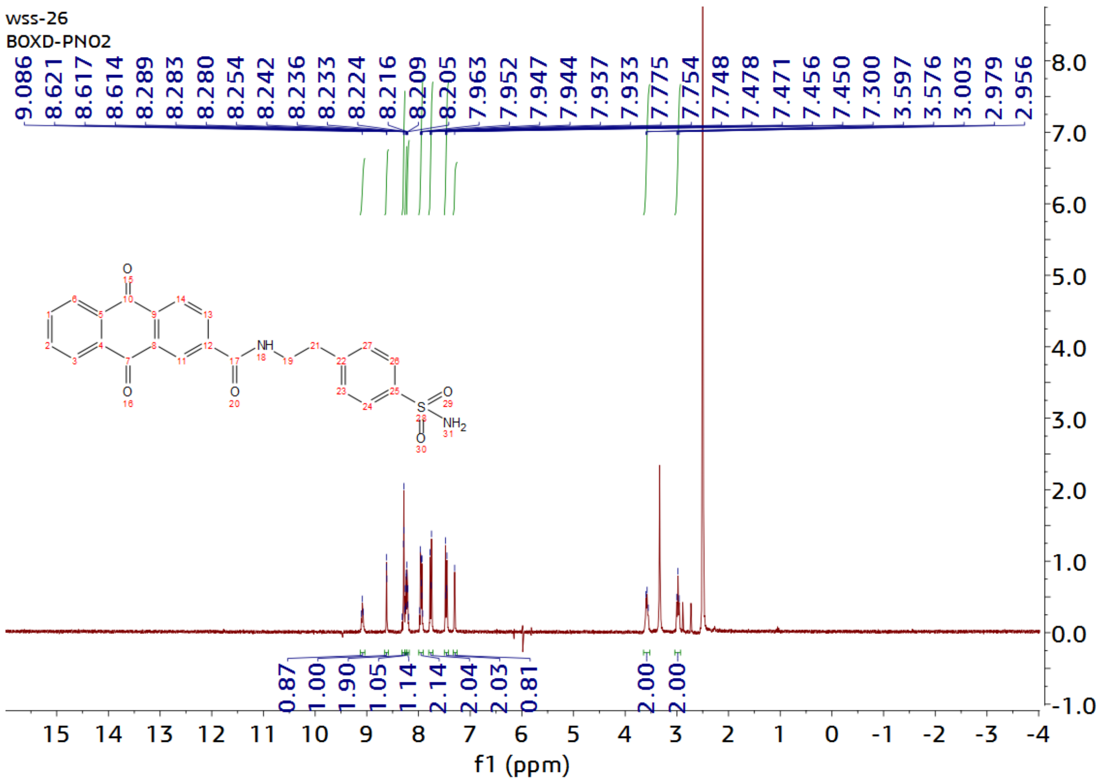

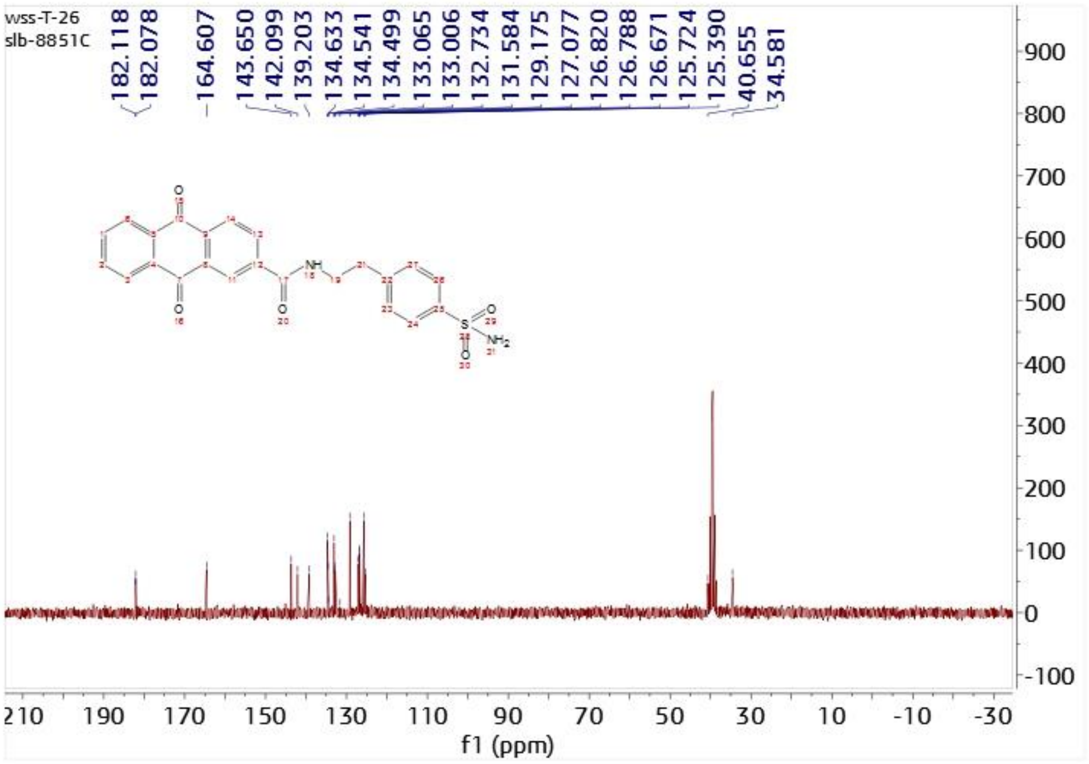

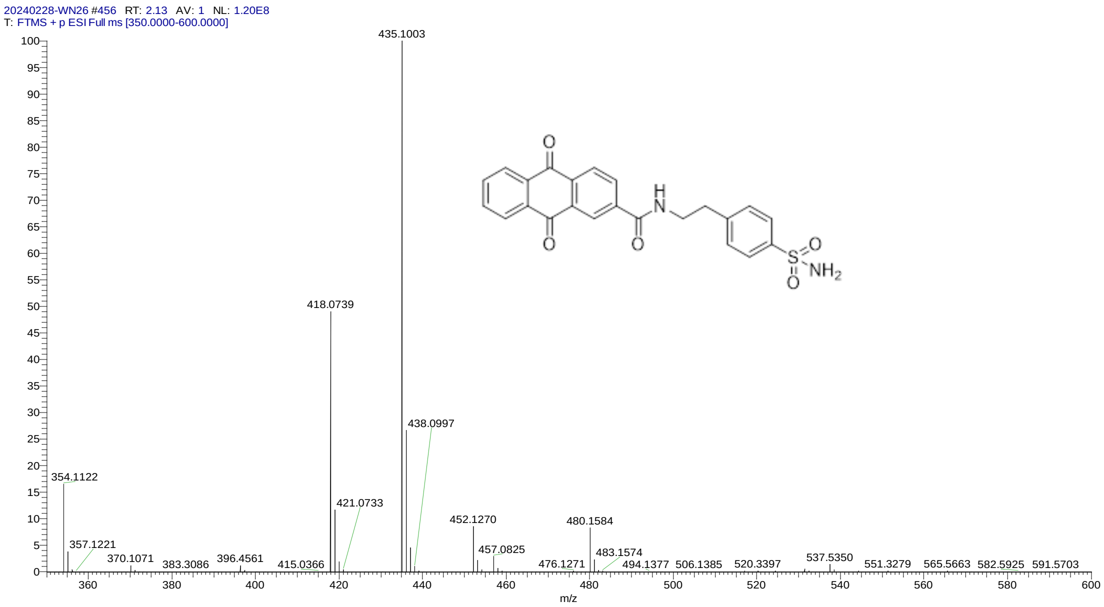

Data S21. ^{1}H-NMR, ^{13}C-NMR and HRMS data of compound 6i

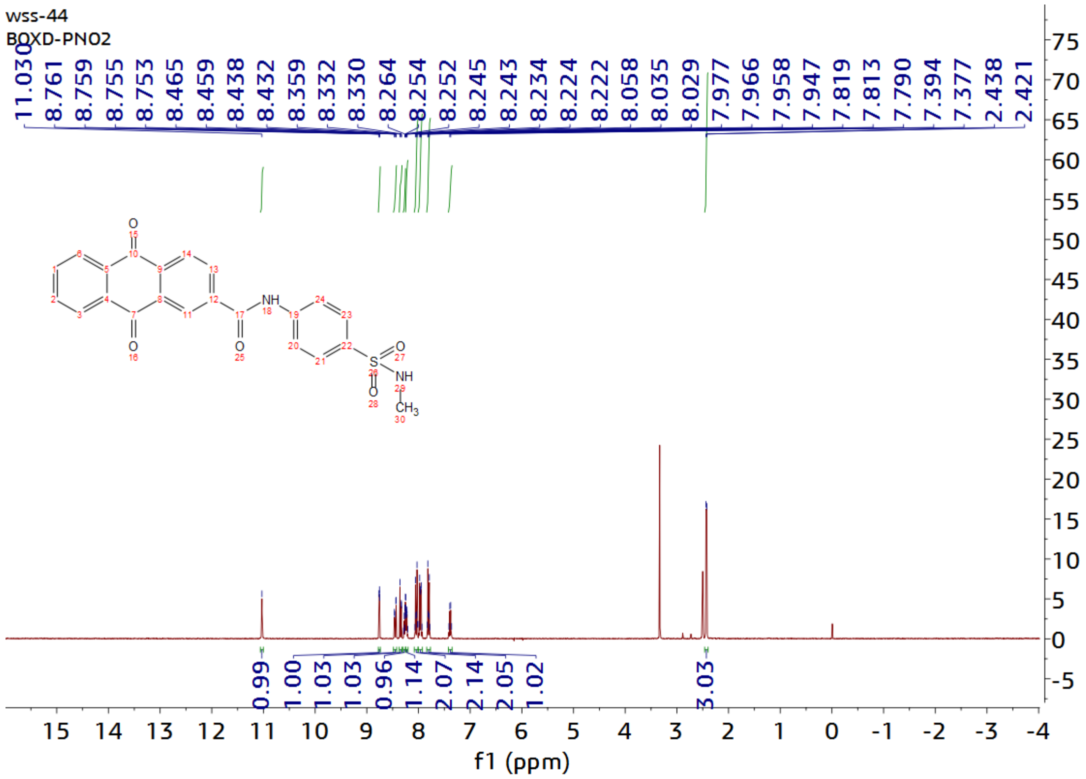
wss-44
BOXD-PNO2
11.030
8.761
8.759
8.755
8.753
8.465
8.459
8.438
8.432
8.359
8.332
8.330
8.264
8.254
8.252
8.245
8.243
8.234
8.224
8.222
8.058
8.035
8.029
7.977
7.966
7.958
7.947
7.819
7.813
7.790
7.394
7.377
2.438
2.421
0.99
1.00
1.03
1.03
0.96
1.14
2.07
2.14
2.05
1.02
3.03
f1 (ppm)

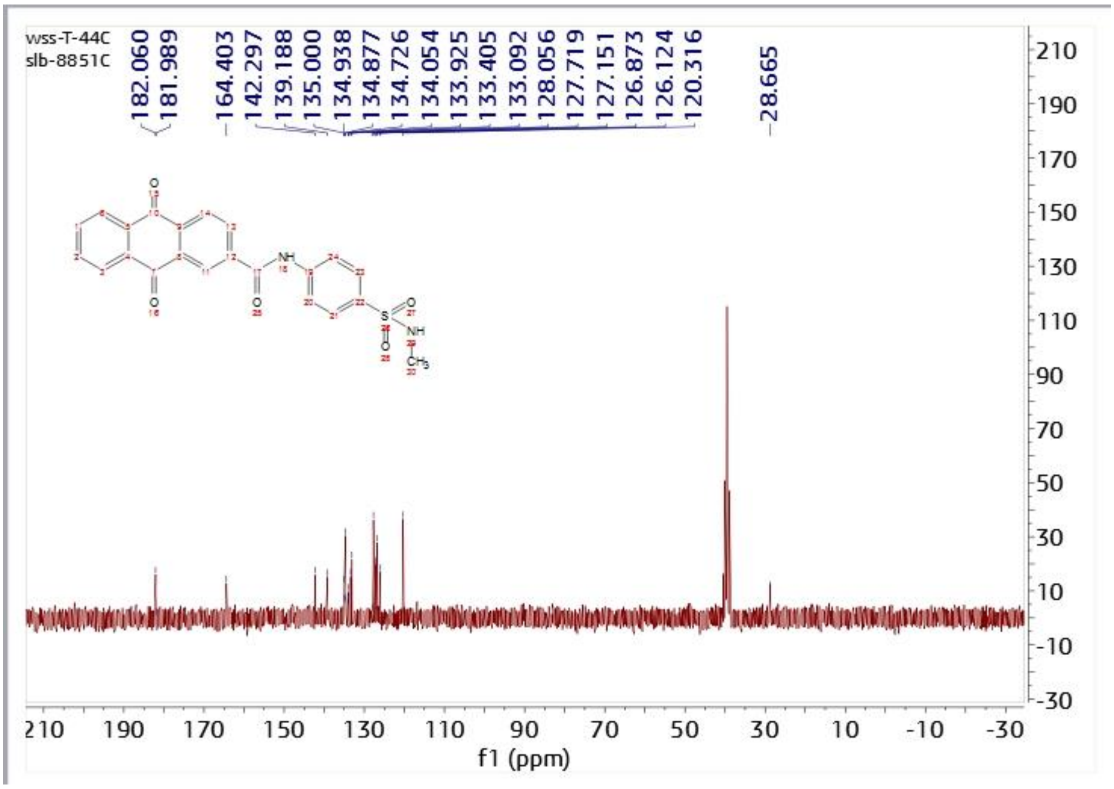
wss-T-44C
slb-8851C
182.060
181.989
164.403
142.297
139.188
135.000
134.938
134.877
134.726
134.054
133.925
133.405
133.092
128.056
127.719
127.151
126.873
126.124
120.316
28.665
f1 (ppm)

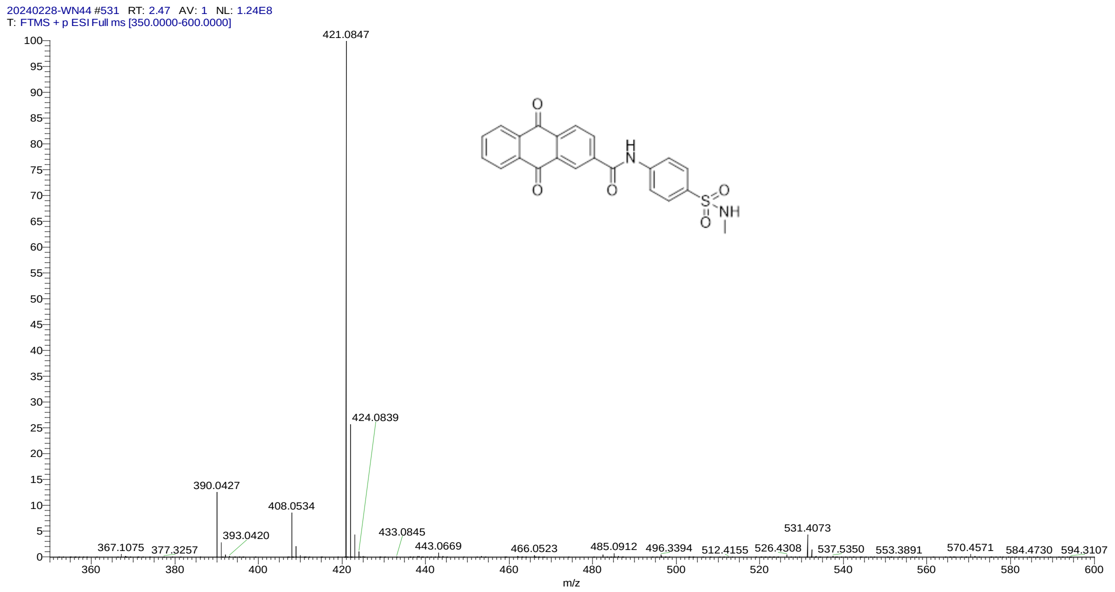

Data S22. ^{1}H-NMR, ^{13}C-NMR and HRMS data of compound 6j

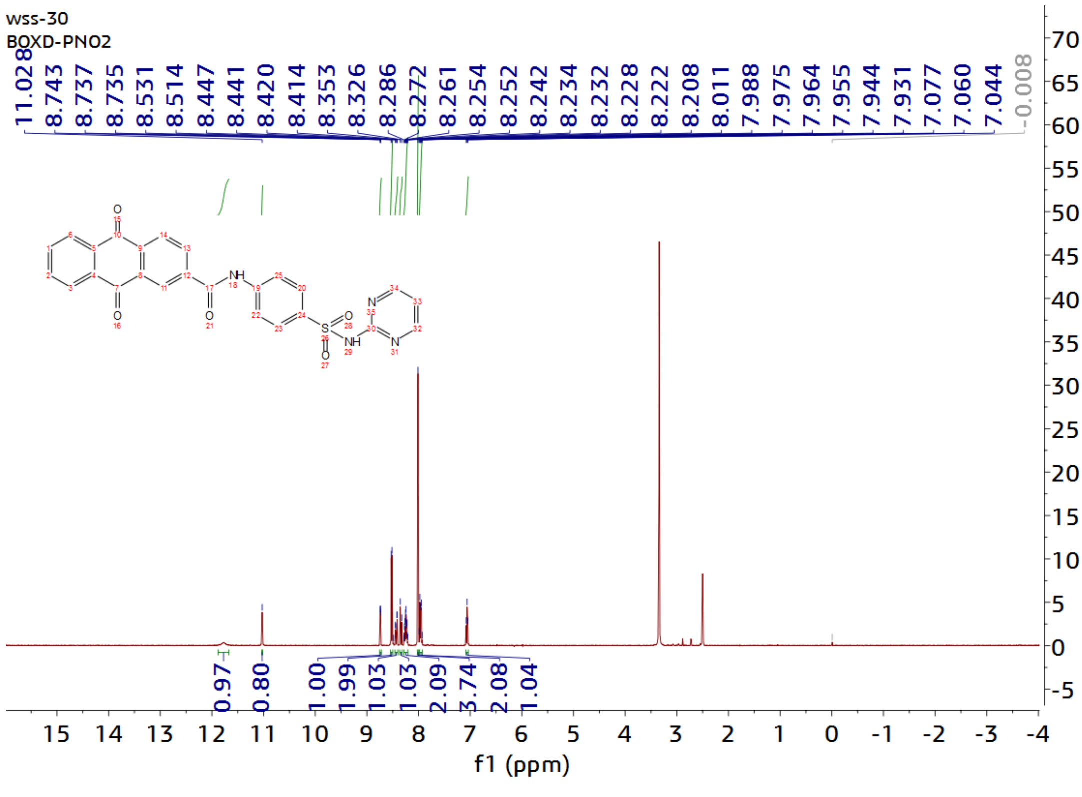

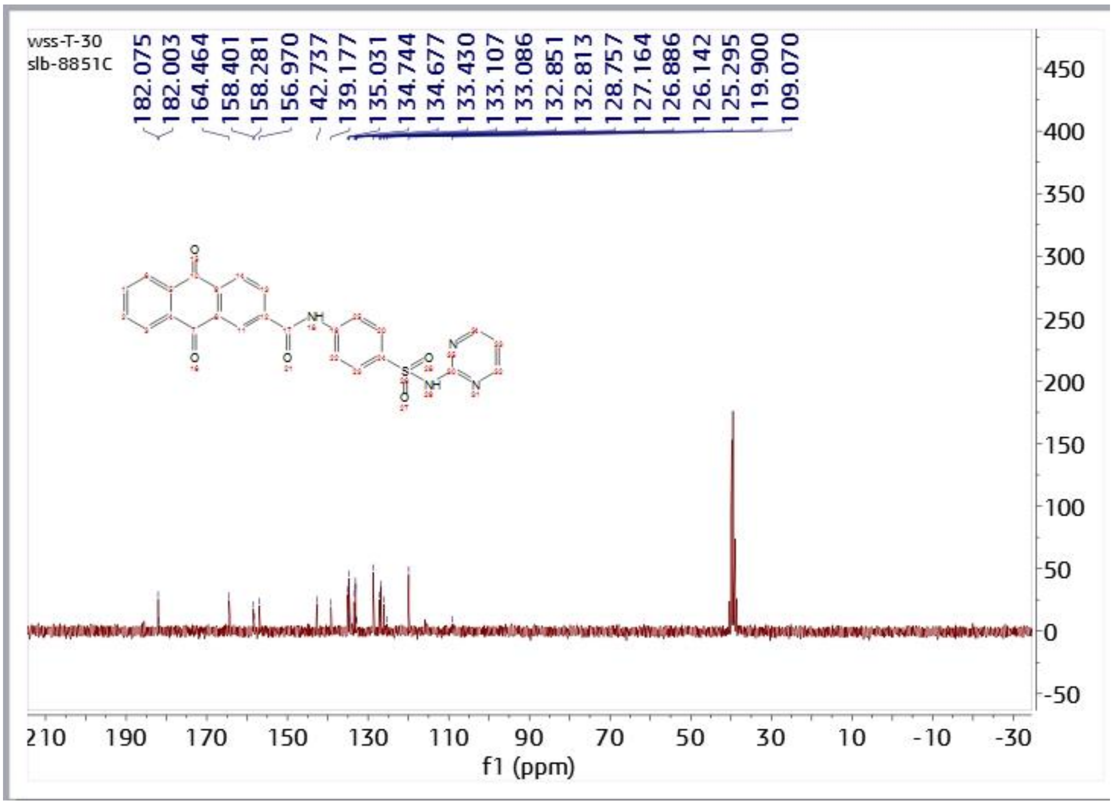

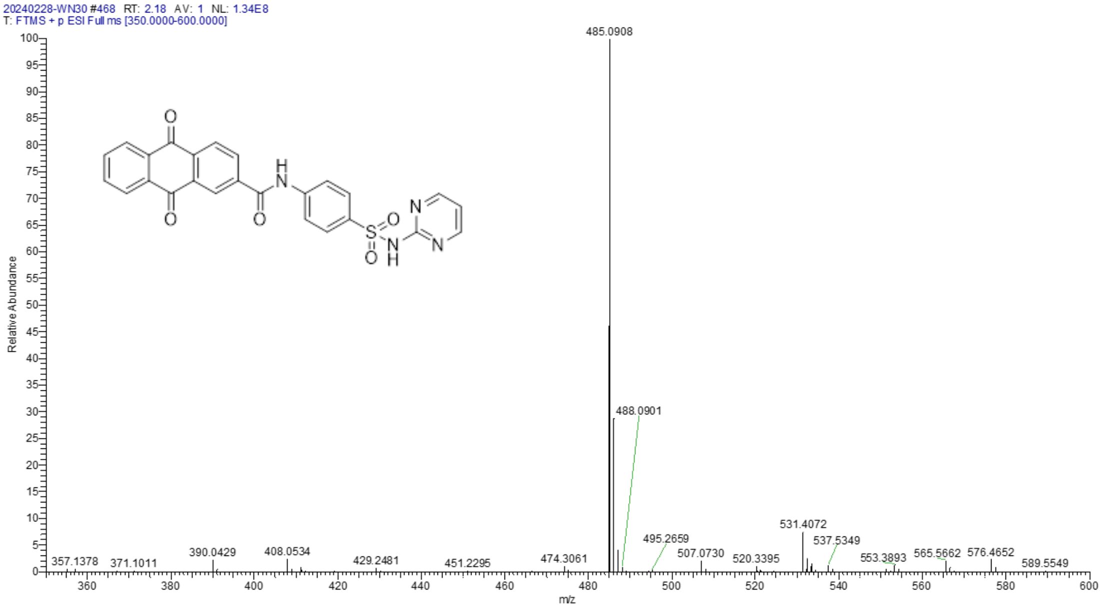

Data S23. ^{1}H-NMR, ^{13}C-NMR and HRMS data of compound 6k

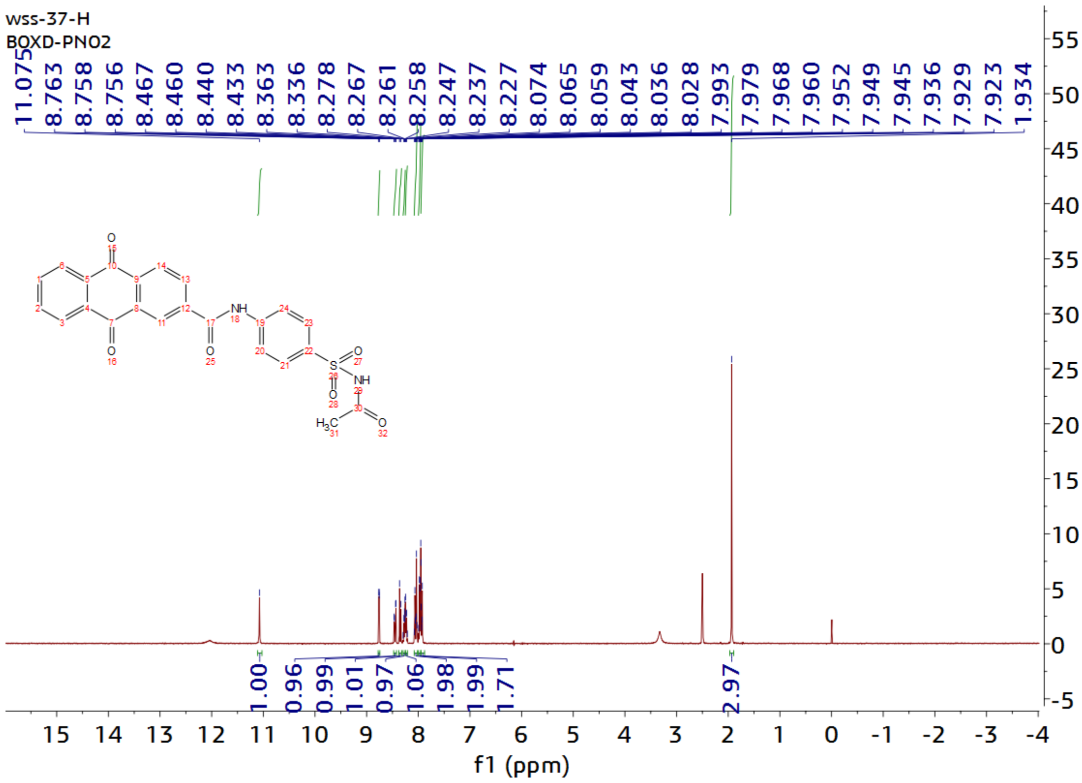
wss-37-H
BOXD-PNO2
11.075
8.763
8.758
8.756
8.467
8.460
8.440
8.433
8.363
8.336
8.278
8.267
8.261
8.258
8.247
8.237
8.227
8.074
8.065
8.059
8.043
8.036
8.028
7.993
7.979
7.968
7.960
7.952
7.949
7.945
7.936
7.929
7.923
1.934
1.00
0.96
0.99
1.01
0.97
1.06
1.98
1.99
1.71
2.97
f1 (ppm)

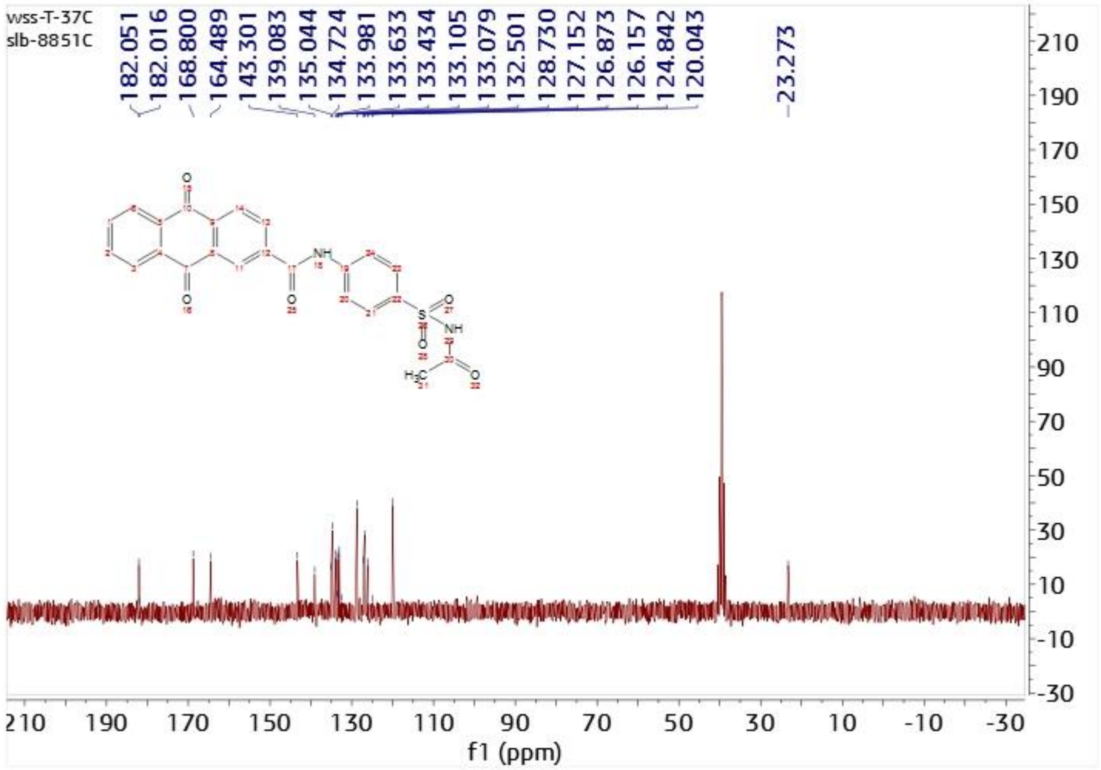
wss-T-37C
slb-8851C
182.051
182.016
168.800
164.489
143.301
139.083
135.044
134.724
133.981
133.633
133.434
133.105
133.079
132.501
128.730
127.152
126.873
126.157
124.842
120.043
23.273
f1 (ppm)

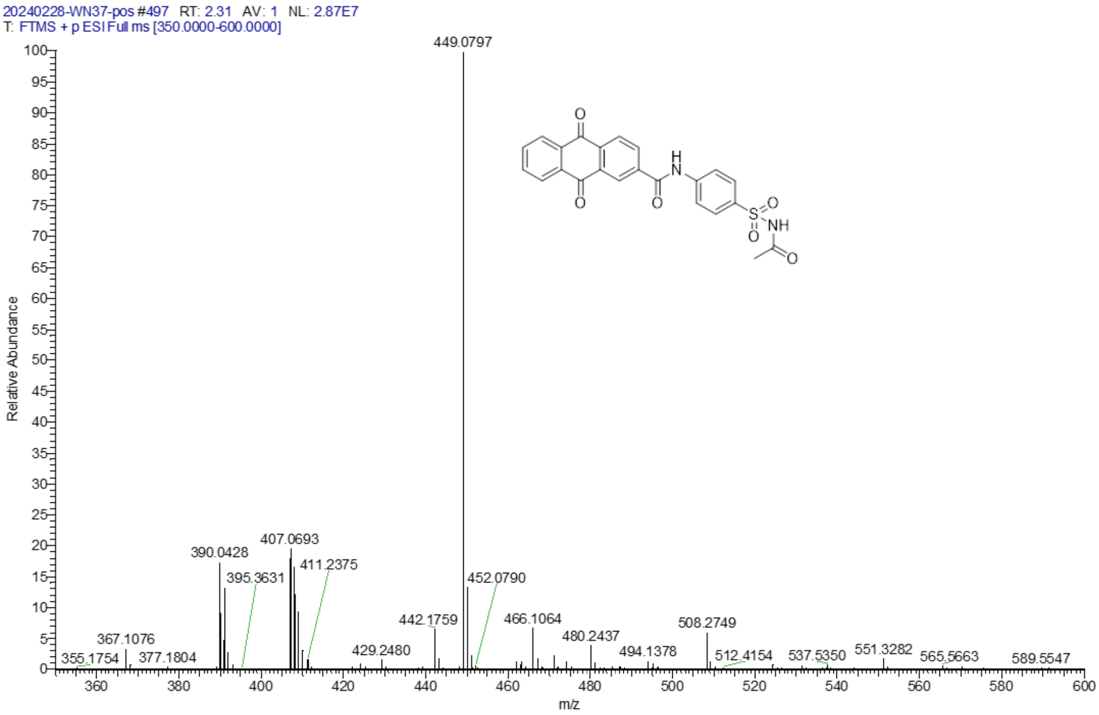

Data S24.HPLC data of compound 5c, 5h, 6c and 6h.

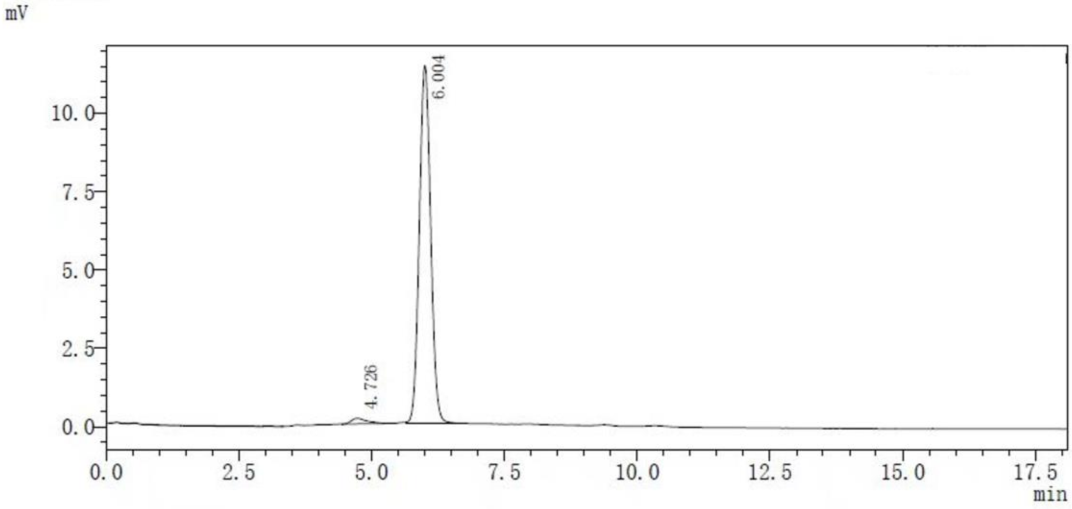

compound 5c

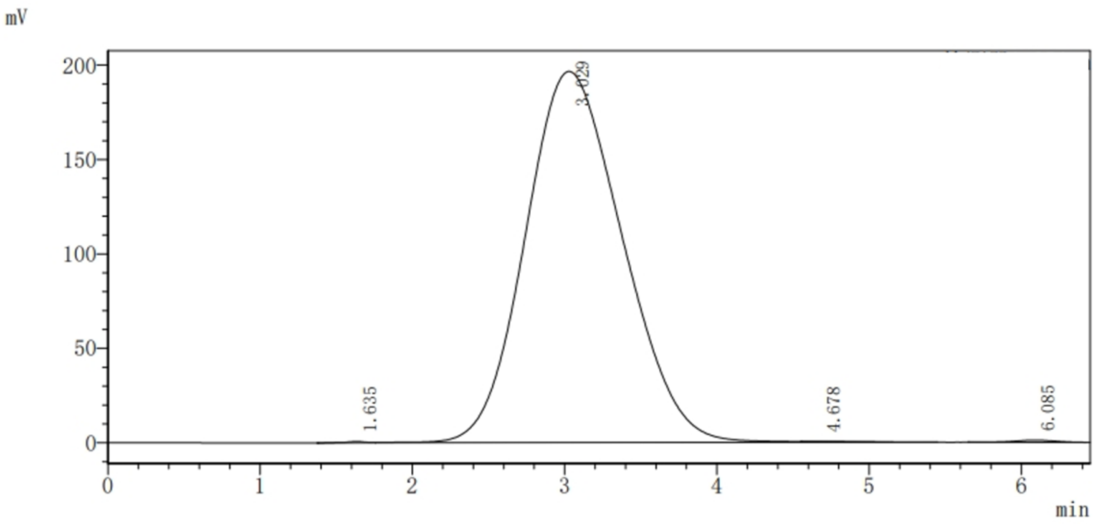

compound 5h

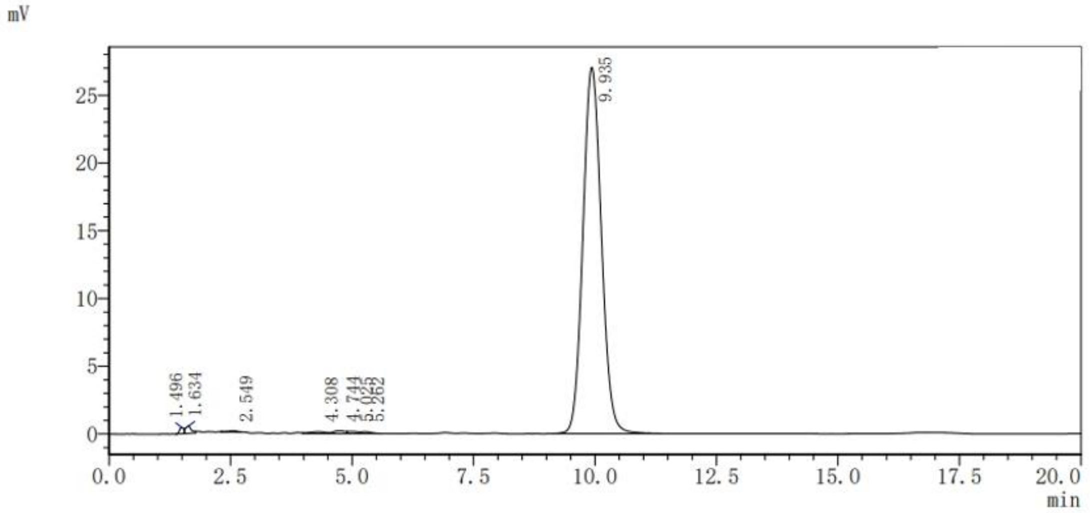

compound 6c

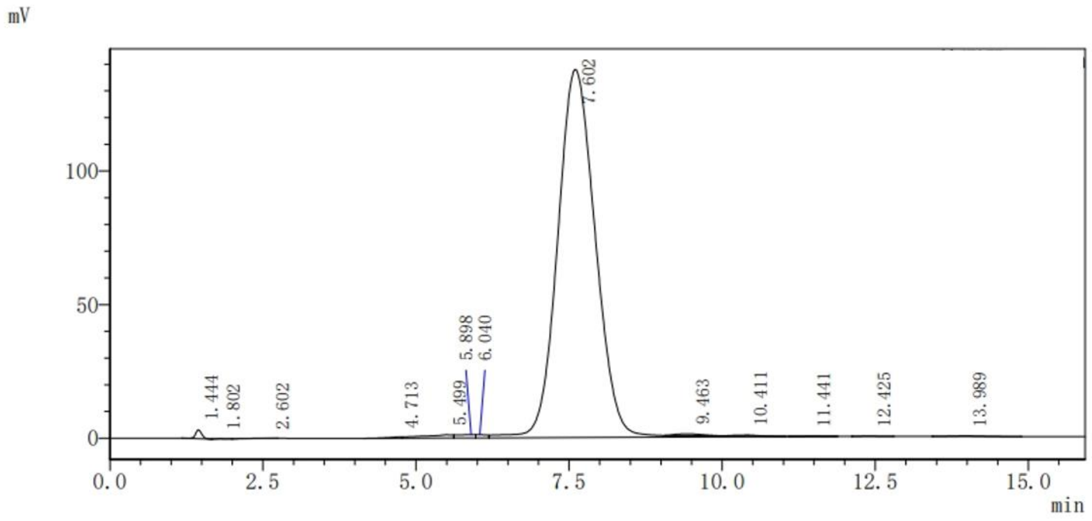

compound 6h
